# The *PIN* gene family in cotton (*Gossypium hirsutum*): genome-wide identification and gene expression analyses during root development and abiotic stress responses

**DOI:** 10.1186/s12864-017-3901-5

**Published:** 2017-07-03

**Authors:** Peng He, Peng Zhao, Limin Wang, Yuzhou Zhang, Xiaosi Wang, Hui Xiao, Jianing Yu, Guanghui Xiao

**Affiliations:** 1 Key Laboratory of the Ministry of Education for Medicinal Plant Resources and Natural Pharmaceutical Chemistry, National Engineering Laboratory for Resource Development of Endangered Crude Drugs in the Northwest of China, College of Life Sciences, Shaanxi Normal University, Xi’an, 710119 China; 2 College of Life Sciences, Shaanxi Normal University, Xi’an, 710119 China; 3 National Key Lab of Crop Genetic Improvement, National Center of Crop Molecular Breeding Technology, National Center of Oil Crop Improvement (Wuhan), College of Plant Science and Technology, Huazhong Agricultural University, Wuhan, 430070 China

**Keywords:** Auxin, PIN-formed, Cotton, Root development, Expression patterns, Abiotic stress

## Abstract

**Background:**

Cell elongation and expansion are significant contributors to plant growth and morphogenesis, and are often regulated by environmental cues and endogenous hormones. Auxin is one of the most important phytohormones involved in the regulation of plant growth and development and plays key roles in plant cell expansion and elongation. Cotton fiber cells are a model system for studying cell elongation due to their large size. Cotton is also the world’s most utilized crop for the production of natural fibers for textile and garment industries, and targeted expression of the IAA biosynthetic gene *iaaM* increased cotton fiber initiation. Polar auxin transport, mediated by PIN and AUX/LAX proteins, plays a central role in the control of auxin distribution. However, very limited information about PIN-FORMED (PIN) efflux carriers in cotton is known.

**Results:**

In this study, 17 PIN-FORMED (PIN) efflux carrier family members were identified in the *Gossypium hirsutum* (*G. hirsutum*) genome. We found that *PIN1–3* and *PIN2* genes originated from the At subgenome were highly expressed in roots. Additionally, evaluation of gene expression patterns indicated that *PIN* genes are differentially induced by various abiotic stresses. Furthermore, we found that the majority of cotton *PIN* genes contained auxin (AuxREs) and salicylic acid (SA) responsive elements in their promoter regions were significantly up-regulated by exogenous hormone treatment.

**Conclusions:**

Our results provide a comprehensive analysis of the *PIN* gene family in *G. hirsutum*, including phylogenetic relationships, chromosomal locations, and gene expression and gene duplication analyses. This study sheds light on the precise roles of *PIN* genes in cotton root development and in adaption to stress responses.

**Electronic supplementary material:**

The online version of this article (doi:10.1186/s12864-017-3901-5) contains supplementary material, which is available to authorized users.

## Background

The plant phytohormone auxin (indole-3-acetic acid, IAA) plays an essential role in plant morphogenesis, organogenesis, apical dominance, embryo formation, vascular differentiation, and light and gravity perception [1, 2]. Two critical pathways, including auxin transport and auxin signaling, are vital for plant development, playing a major role in both phototropism and gravitropism. At the cellular level, auxin negatively regulates the transcription factor AUXIN RESPONSE FACTOR (ARF), which mediates the expression of auxin-responsive genes [3, 4]. At the tissue level, auxin is synthesized and then transported from the site of biosynthesis to the sites of auxin action [5, 6]. Auxin efflux has been observed in specific tissues at different developmental stages, and plays a role in lateral organ initiation, root gravitropism, and root hair formation [7–9]. The cell to cell polar transport of auxin is mediated by specific influx and efflux carriers, resulting in asymmetric distribution of auxin [10, 11]. The auxin influx and efflux proteins in *Arabidopsis* can be grouped into 3 gene families: P-glycoprotein (MDR/PGP/ABCB) efflux/conditional transporters (PGP), auxin resistant 1/like aux1 (AUX1/LAX) influx carriers, and plant specific PIN-FORMED (PIN) efflux carriers [4, 12, 13]. The polar subcellular localization of PIN efflux proteins is responsible for directional auxin flow, and more and more evidence indicates that plasma membrane-localized PIN proteins are the rate-limiting step in auxin polar transport [14, 15]. PIN polarization and auxin polar transport play an important role in both plant phototropism and gravitropism [16, 17].

In *Arabidopsis*, the *PIN* gene family is comprised of 8 members, which have been reported to be involved in various developmental processes [18, 19]. PIN1, PIN2, PIN3, PIN14 and PIN7, located in the plasma membrane, are involved in the tropic response and root growth. In contrast, the endoplasmic reticulum (ER) localized PINs, including PIN5, PIN6 and PIN8, are mainly involved in intracellular auxin homeostasis [20, 21]. Among all PIN family members, loss-of-function *pin1* and *pin2* mutants show severe phenotypes, making them well suited for investigating auxin-dependent developmental processes [22]. PIN1 is mainly involved in the maintenance of embryonic auxin gradients, and organ initiation is severely affected in *pin1* mutants, which results in the formation of naked inflorescence stems [23]. Constitutive triple response 1 (CTR1), a protein that acts downstream of the ethylene receptors, is a negative regulator of ethylene signaling. *PIN2* functions downstream of CTR1 and primarily regulates root gravitropism [24]. The roots of *pin2* mutants are insensitive to ethylene and grow agravitropically [25, 26]. Recently, the *PIN* genes have been reported to play a role in the integration of hormone signaling and abiotic stress responses. In soybean, *PIN* genes are differentially regulated by both abiotic stresses and phytohormones [19]. In rice, *PIN* genes show tissue-specific expression and have been found to also be regulated by hormones [27]. In maize, the expression levels of most *ZmPIN* genes were induced in shoots and reduced in roots by various abiotic stress treatments, including drought, salt, dehydration and cold [15].

Cotton is an important source of both natural fibers in the textile industry and cotton seed oil used in the production of food and biodiesel fuel. Understanding the factors that regulate fiber initiation will drive the development of technologies to improve yield potentials. Prior work suggests that auxin plays an essential role in fiber cell initiation. Exogenous application of IAA to cotton ovules promoted cotton fiber initials during fiber cell initiation [28]. Furthermore, in vitro application of N-1-naphthylphtha-lamic acid (NPA), an inhibitor of auxin polar transport, reduces IAA accumulation and inhibits fiber cell initiation [29]. Further, targeted expression of *iaaM*, an IAA biosynthetic gene, was showed to increase the number of lint fibers produced [29]. In additional, auxin regulates cotton fiber initiation via GhPIN-mediated auxin transport [30, 31]. Auxin is not synthesized in fiber cells, and is mainly transported from the outside of ovules to fiber cells via polar auxin transport.

In this work, we performed a comprehensive analysis of the *PIN* gene family based on data gathered from recent whole genome sequencing results [32–36]. Comparative analysis of allotetraploid cotton (*G. hirsutum*) with its diploid ancestor (*G. arboreum*) indicated that *PIN1–3* and *PIN2* may play an important role in root development. In addition, we carried out expression profiling of cotton *PIN* genes in response to different hormonal treatments and abiotic stresses. The results showed that the majority of cotton *PIN* genes contained auxin response elements (AuxREs) and salicylic acid (SA) responsive elements in their promoter regions, which were significantly up-regulated by exogenous hormone treatment.

## Methods

### Plant materials and growth conditions

*Gossypium hirsutum* (Xuzhou 142) and *Gossypium arboreum* (Shixiya 1) seeds were acquired from the Institute of Cotton Research of the Chinese Academy of Agricultural Sciences (Anyang, China). The seeds were planted into sand containers (one seedling per container) and grown in a climate-controlled greenhouse (16-h light and 8-h dark cycle at 30 °C) located at Shaanxi Normal University. For each assay, samples were collected from the equivalent growth stages of *G. hirsutum* and *G. arboreum*. A total of 9 seedlings of each species were used for each treatment and three biological triplicates were performed per assay.

### Identification and phylogenetic analysis of cotton *PIN* genes

Genome sequences of *G. hisutum*, *G. arboreum* and *G. barbadense* have recently become available [32–38]. A genome browser for the cotton genome, termed Cottongen, is accessible online (https://www.cottongen.org). The *A. thaliana* genome sequence was acquired from TAIR 10 (http://www.arabidopsis.org). Putative cotton PINs were identified by blast searches against the three reference genomes using *A. thaliana* PIN protein sequences as queries. Next, candidate PINs were further filtered based on their conserved domains using SMART (http://smart.embl-heidelberg.de) and Pfam (http://pfam.xfam.org/search#tabview=tab0) database analyses [39, 40]. The phylogenetic relationships between putative cotton PINs and *A. thaliana* PIN proteins were determined using the neighbor-joining algorithm using default parameters with 1000 bootstrap analyses by MEGA 5.0 (https://www.megasoftware.net). Based on the phylogenetic analysis, the putative cotton PINs were named according to their respective clades. Multiple sequence alignments of all newly identified PINs in this study were performed using ClustalX with default parameters [41].

### Chromosomal location and gene duplication analysis

Chromosome position information of the putative PINs in *G. hirsutum* was obtained from gene annotation files downloaded from the CottonGen website (https://www.cottongen.org). The map of *PIN* gene distribution along the chromosomes is shown from top to bottom [42]. Duplication analysis of *G. hirsutum PIN* genes and genomic synteny was identified and displayed with the Synteny Mapping and Analysis Program (SyMAP) v3.4 [43].

### Stress and hormonal treatments

Plants used for stress and hormone treatments were grown under the same greenhouse conditions as reported previously [44]. The same protocols were used for drought, dehydration and salt (250 mM) abiotic stress treatments [44]. For the plant hormone treatments, the methods were modified from the methods utilized by Chai et al. [45]. Briefly, two-week old seedlings were irrigated and sprayed with 10 μM NAA and 0.5 mM salicylic acid, respectively. After treatment, root and shoot tissues were harvested at indicated times (0.5, 1, 3 and 5 h). The samples were frozen in liquid nitrogen immediately after collection and kept at −80 °C. For each treatment, three individual samples were collected and analysis was performed on biological triplicates.

### RNA extraction and quantitative RT-PCR (qRT-PCR) analysis

Cotton samples were ground to fine powder with a mortar and pestle in liquid nitrogen. Total RNA was isolated using a modified CTAB method as described [46], and 5 μg of total RNA was converted into cDNA using the cDNA Synthesis SuperMix (Transgen, China) according to the manufacture instructions. For qRT-PCR experiments, cotton *UBQ7* (GenBank No. AY189972) was used as an internal control. Primers for qRT-PCR analysis are listed in Additional files 1: Table S4.

### Analysis of regulatory elements in the promoter region

Identified *GhPIN* genes including their predicted promoter sequences were downloaded from the CottonGen website (https://www.cottongen.org). The regulatory elements in the promoter regions were predicted using PLACE and PlantCARE software as previously [47, 48].

### Accession numbers

The TAIR accession numbers for the *Arabidopsis* PIN sequences used in this study are as follows: At1g73590 (*AtPIN1*), At5g57090 (*AtPIN2*), At1g70940 (*AtPIN3*), At2g01420 (*AtPIN4*), At5g16530 (*AtPIN5*), At1g77110 (*AtPIN6*), At1g23080 (*AtPIN7*), At5g15100 (*AtPIN8*).

## Results

### Genome-wide identification of PIN proteins in cotton

Three cotton genomes, including two diploid (*G. arboreum* and *G. raimondii*) and one allotetraploid (*G. hirsutum*), have recently been completed and published [33–36]. A genome browser is available at the Cottongen website (https://www.cottongen.org). Putative cotton PIN proteins were initially identified by Blastp searches against the three reference genomes using *A. thaliana* PIN protein sequences as queries. After protein conserved domain and gene structure selection, a total of 17, 12, and 10 predicted protein coding sequences (CDSs) were identified in *G. hirsutum*, *G. arboreum* and *G. raimondii*, respectively (Additional file 2: Table S1). Most *G. hirsutum PIN* genes contained at least 3 introns in their open reading frames, except *PIN1–4-D* and *PIN8–2-A*, which have only one intron and two introns, respectively. The protein coding sequences of all *GhPINs* are listed in Additional file 3: Table S2. The deduced GhPIN proteins varied in length from 127 amino acids (GhPIN8–2-A) to 678 amino acids (GhPIN2-D). Conserved domain analysis showed they have an auxin efflux carrier (ACE) domain at the amino-terminal region and a membrane transport (MT) domain at the carboxy-terminus (Additional file 4: Figure S1). Although the predicted sizes of the putative GhPIN proteins varied markedly, these two domains were found to be consistently present in all analyzed sequences, indicating that these two domains are likely to be crucial for biochemical function.

### Phylogenetic analysis and chromosomal distribution of *GhPIN* genes

**Fig. 1:**
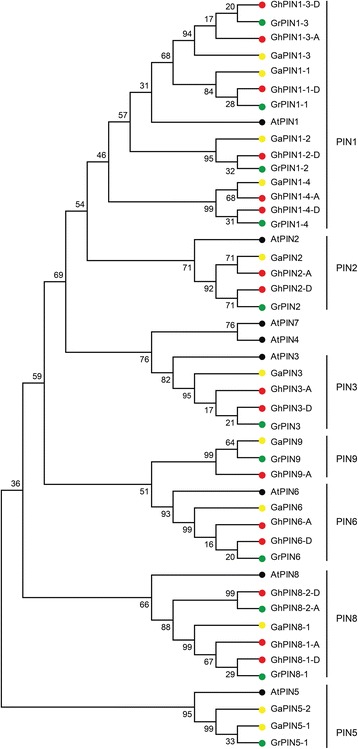
Phylogenetic analysis of *PIN* family genes from *G. hirsutum*, *G. arboreum*, *G. raimondii* and *A. thaliana.* The phylogenetic tree was constructed using MEGA 5.0 with the neighbor-joining method. The origin of the *PIN* genes are indicated by colored circles as follows: *G. hirsutum* (*red*), *G. arboretum* (*yellow*), *G. raimondii* (*green*), and *A. thaliana* (*black*). Numbers on branches are bootstrap values calculated from 1000 replicates

In order to investigate the evolutionary relationships of the identified PIN proteins, an unrooted phylogenetic tree was generated using the predicted full length amino acid sequences from *G. arboreum*, *G. raimondii*, *G. hirsutum* and *A. thaliana*. As illustrated in the Neiboring-Joining phylogenetic tree (Fig. 1), a similar organization for the cotton and *A. thaliana* PIN proteins and some orthologous relationships between both species were identified. Based on this analysis, cotton PIN proteins were named based on their relationships to known *A. thaliana* PINs. Compared with *A. thaliana*, the *PIN1* subfamily was found to be extensively expanded in cotton, indicating they may play an important role in cotton. Interestingly, *PIN5* subfamily genes were found in *G. arboreum*, *G. raimondii* and *A. thaliana*, but all of them were lost in *G. hirsutum* and *G. barbadense* (Additional file 5: Table S3), implying that a gene loss event occurred in the *PIN* family genes after polyploidization in the allotetraploid cotton.

**Fig. 2:**
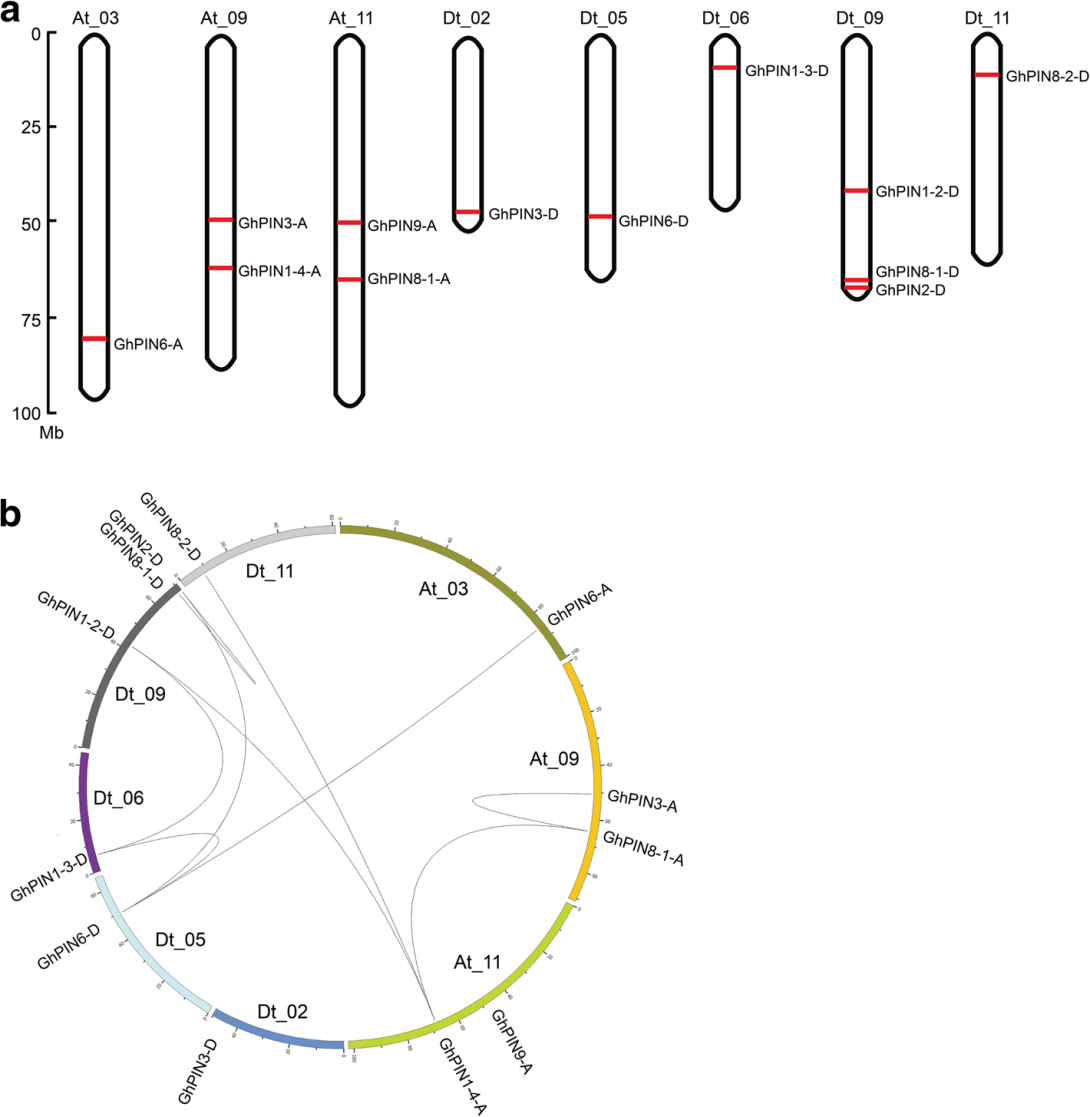
Chromosomal location of the identified *GhPIN* genes in *G. hirsutum*. **a** Chromosomal location of the 12 mapped *GhPIN* genes is depicted from top to bottom. The *G. hirsutum* chromosomes have been divided into At and Dt chromosomes according to the *G. hirsutum* genome. The scale bar is in mega bases (Mb). Chromosome numbers are indicated on the top of the corresponding chromosomes. **b** Analysis of *GhPIN* gene duplication. The genome visualization tool CIRCOS was used. Black lines represent duplicated gene pairs

*GhPIN* genes were mapped onto chromosomes to provide insights into their organization in the cotton genome. This data indicated that 12 genes were distributed among 8 chromosomes, including 5 *GhPIN* genes on 3 chromosomes from the At-subgenome and 7 genes found on 5 chromosomes from the Dt-subgenome (Fig. 2a). Two whole-genome duplication (WGD) events have been observed in cotton [36], which resulted in the occurrence of gene duplication. The relationships and duplication events between the 12 *PIN* genes were investigated. These combined data suggested that segmental duplication and dispersed duplication might be the main types of *PIN* gene duplication in cotton (Fig. 2b).

### *GhPIN1–3* and *GhPIN2* are required for cotton root development

**Fig. 3:**
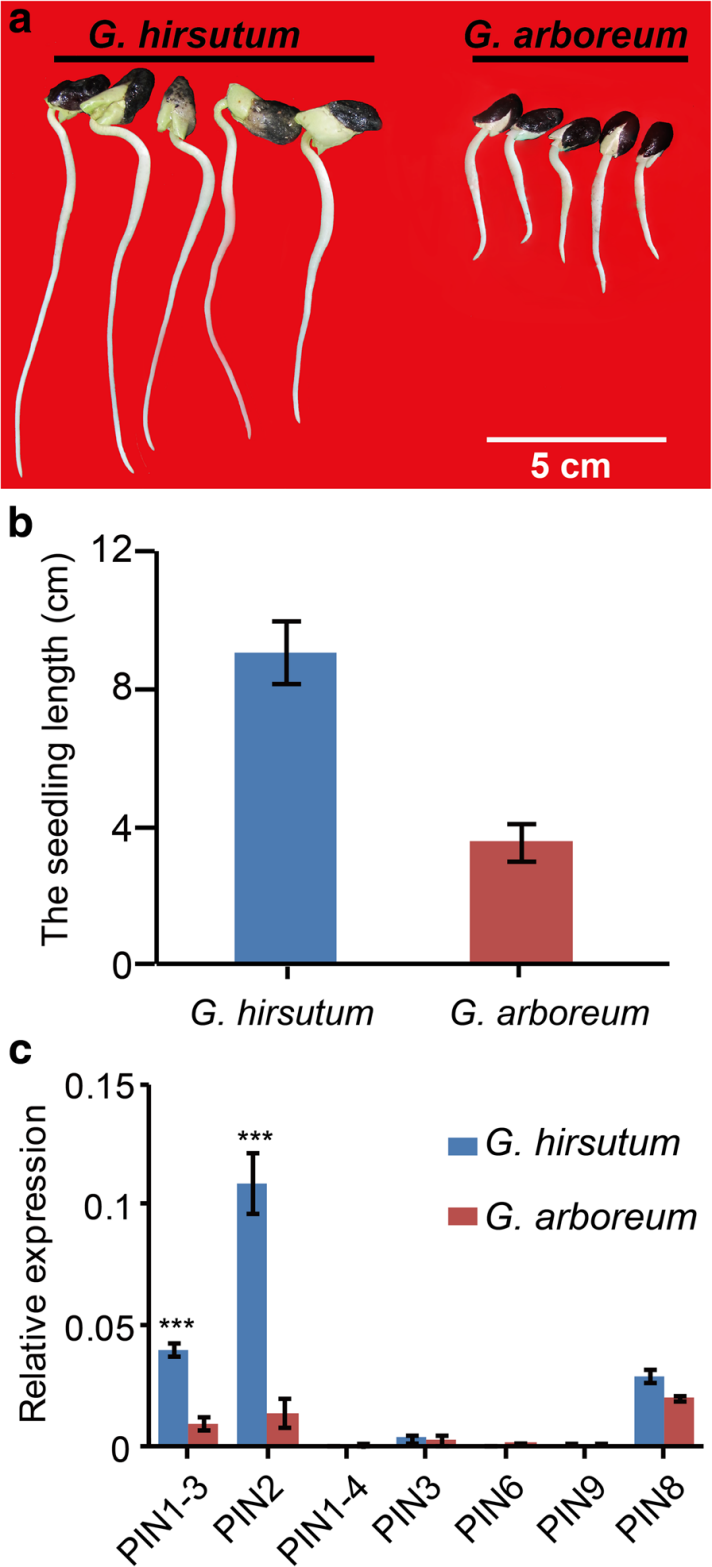
Comparative analysis of root growth in *G. hirsutum* and *G. arboreum*. **a** Representative roots from 7-d-old *G. hirsutum* and *G. arboreum* seedlings. Bar = 5 cm. **b** Root lengths of 7-d-old *G. hirsutum* and *G. arboreum* seedlings as shown in (**a**). Statistical analysis was carried out on three independent experiments. A total of 10 seedlings were used for each measurement. Root lengths are shown as means ± SE. **c** Quantitative RT-PCR analysis of *PIN* genes in 7-d-old *G. hirsutum* and *G. arboreum* roots

**Fig. 4:**
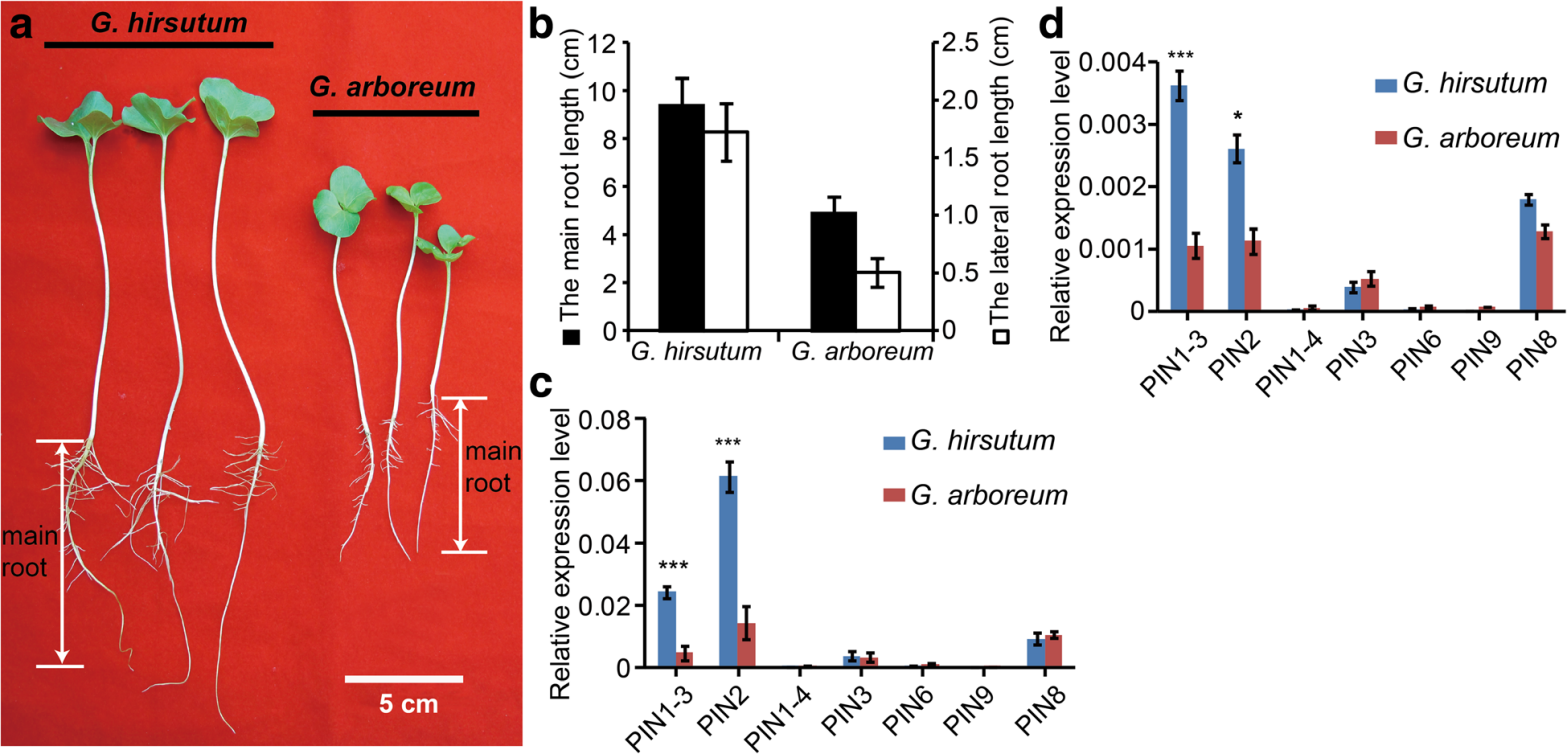
The development of main and lateral roots in *G. hirsutum* and *G. arboreum*. **a** Comparisons of main and lateral root lengths of three-week-old *G. hirsutum* and *G. arboreum* seedlings. Bar = 5 cm. **b** Measurements of main and lateral root lengths of *G. hirsutum* and *G. arboreum* seedlings as shown in (**a**). Statistical analyses were carried out on three independent experiments. A total of 9 seedlings were used for each measurement. The lateral root length in this figure represents the average lengths of all investigated lateral roots. Root lengths are shown as means ± SE. Closed bars with scales to their left side and open bars with scales to their right side indicate the main root lengths and the lateral root lengths, respectively. **c**, **d** The relative expression of *PIN* genes in main (**c**) and lateral roots (**d**) from three week old plants, qRT-PCR experiments were performed on three biological replicates. Error bars represent means ± SE from three independent experiments. The relative expression level was determined using cotton *UBQ7* as a control

Roots play an essential role in plant growth by absorbing water and inorganic nutrients from the ground. Auxin, which is transported and regulated by auxin efflux transporters, has been reported as a positional cue for root cell type determination [49]. To evaluate the roles of *PIN* genes in cotton root development, a comparative analysis of root growth between *G. hirsutum* and *G. arboretum* was carried out. 7 days after germination, a significant difference in root length was observed between *G. hirsutum* and *G. arboreum* (Fig. 3a). The roots of *G. hirsutum* reached an average length of 8 cm, while roots of *G. arboreum* were less than 4 cm (Fig. 3b). Next, we tested the expression of *GhPIN* genes during root development. qRT-PCR results showed that the expression levels of *GhPIN1–3* and *GhPIN2* in *G. hirsutum* were much higher than their predicted orthologs in *G. arboreum* (Fig. 3c), indicating that *GhPIN1–3* and *GhPIN2* genes may be required for early root development. After 3 weeks, both the main and lateral roots of *G. hirsutum* were significantly longer than those of *G. arboreum* (Fig. 4a and b). However, no difference in the number of lateral roots was observed between *G. hirsutum* and *G. arboreum* (Additional file 6: Figure S2). Among the 7 *PIN* genes analyzed, *GhPIN1–3* and *GhPIN2* were found to be higher expression in both main and lateral roots of *G. hirsutum* relative to *G. arboreum* (Fig. 4c and d), indicating that *GhPIN1–3* and *GhPIN2* may play key roles in regulating the late-stage development of cotton roots. Furthermore, the expression data of *GhPIN2* suggests that it may contribute more than *GhPIN1–3* in regulating cotton root development (Figs. 3c and 4c). However, *GhPIN1–3* was found to be more highly expressed in lateral roots, suggesting that it plays more an important role in regulating lateral root growth (Fig. 4d). The observed differences in gene expression during root development indicate possible functional differentiation of *PIN* genes.

### Expression of *GhPINs* in response to drought, salt and dehydration

**Fig. 5:**
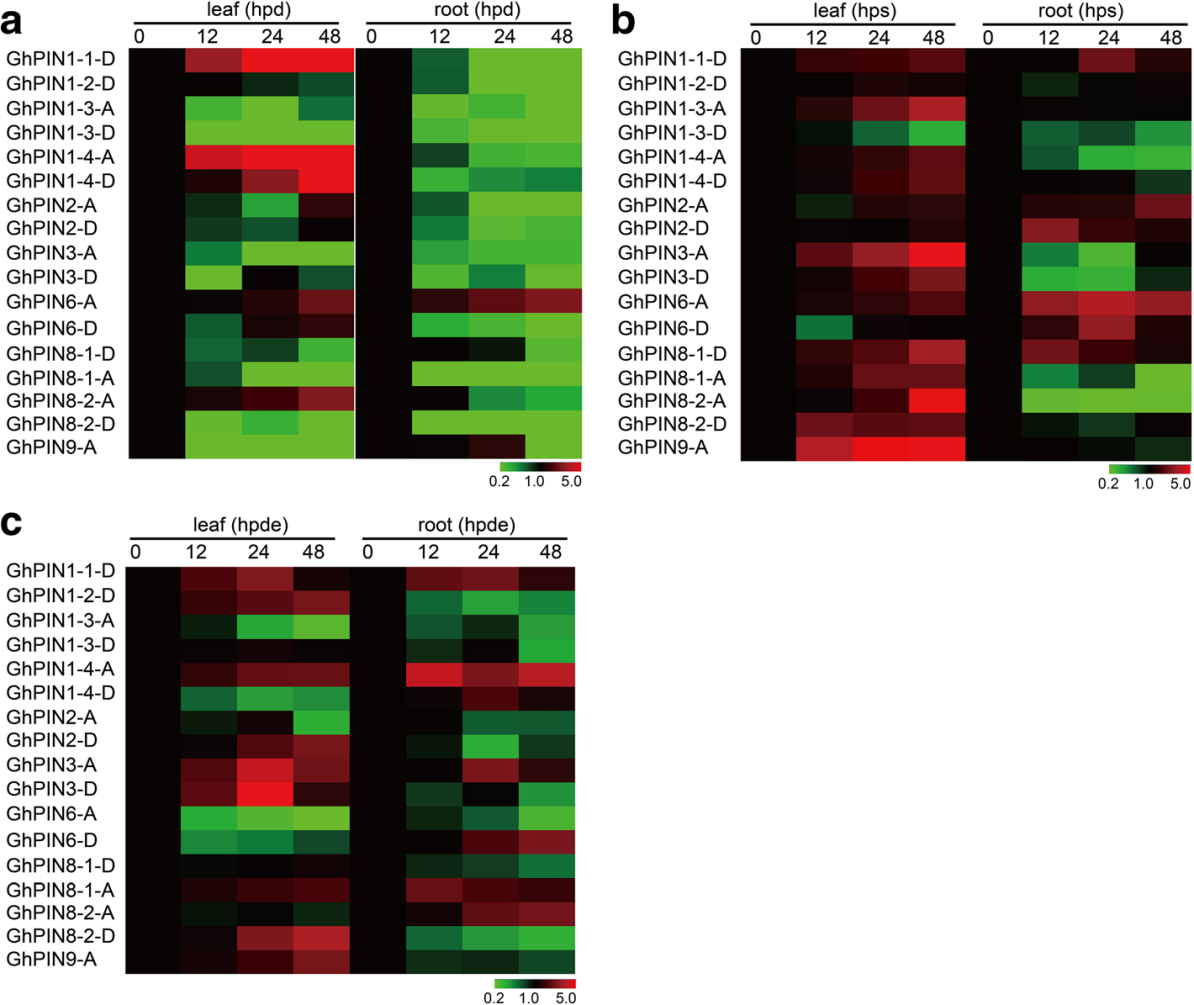
Gene expression analysis of *GhPINs* after abiotic stresses treatments. **a** Fold changes of *GhPIN* gene expression under drought treatment. **b** Fold changes of *GhPIN* gene expression under salt treatment. **c** Fold changes of *GhPIN* gene expression under dehydration treatment. All treatments were performed with three biological and three technical replicates. Hpd, hours post drought; hps, hours post salt; hpde, hours post dehydration. The relative gene expression levels were determined using cotton *UBQ7* as a control

It has been reported that *PIN* genes are involved in plant response to diverse abiotic stresses, such as drought, salt and dehydration [50]. In order to identify potential roles of individual cotton *PIN* genes in different abiotic stress responses, quantitative real-time PCR (qRT-PCR) analysis was performed to identify changes in gene expression in leaves and roots of plants that underwent different stress treatments. *GhPIN1–1-D* and *GhPIN1–4-A* were found to be highly differentially expressed. More *GhPINs* were up-regulated in both leaves and roots that underwent salt and dehydration treatment, relative to plants subjected to drought stress. After cotton seedlings were grown in drought conditions for 48 h, 5 genes were induced in leaves (list genes) while only *GhPIN6-A* was up-regulated in roots (Fig. 5a). *GhPIN1–1-D, GhPIN1–4-A* and *GhPIN1–4-D* were highly upregulated in leaves under drought stress, but concomitantly down-regulated in roots. More genes showed significant changes in gene expression in leaves, when compared to roots, and almost all genes (13 out of 17) were highly expressed in leaves in plants grown in saline conditions (Fig. 5b). Interestingly, the number of *GhPIN* genes up-regulated in leaves and roots were nearly identical after drought stress (Fig. 5c). In general, *GhPIN* genes were widely induced by drought, salt and dehydration stresses, suggesting that *GhPINs* play a role in abiotic stress responses in cotton.

### Gene expression analysis of *GhPINs* in response to auxin and salicylic acid treatment

**Fig. 6:**
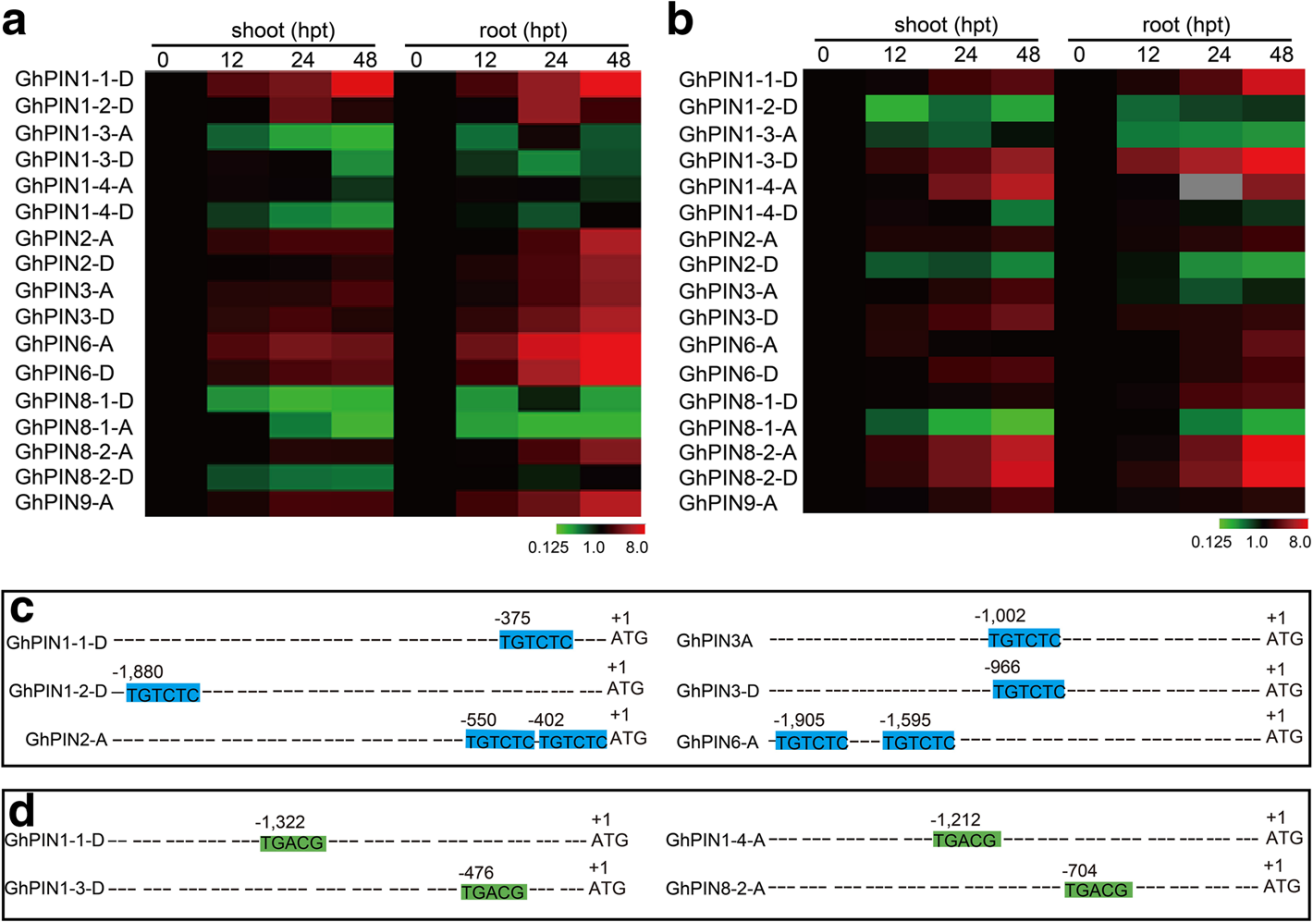
Quantitative RT-PCR analysis of *GhPINs* form roots and shoots of cotton plants treated with auxin or salicylic acid. **a** Expression of *GhPIN* genes after NAA treatment. **b** Transcript abundance of *GhPIN* genes under salicylic acid treatment. Hpt indicates hours post treatment in (**a**) and (**b**). **c** Analysis of *GhPINs* with AuxRE elements present in their promoter regions. The element (TGTCTC) is the putative ARF binding site, which is one of the key auxin responsive elements (AuxREs). **d** Analysis of *GhPINs* with SA responsive elements present in their promoter regions. The element (TGACG) is the putative SA responsive element

The phytohormone auxin is known to play important roles in plant development [51]. Emerging evidence indicates that salicylic acid (SA), a pivotal signaling molecule involved in plant immune responses, is involved in both local and systemic disease resistance responses [52]. In order to explore the effects of auxin and SA on *GhPINs* expression, *GhPIN* gene expression analysis was carried out on shoots and roots after treatment with plant hormones were analyzed. *GhPINs* were differentially expressed upon treatment with SA and the auxin analogue NAA,. The results showed that a total of 8 and 10 *GhPIN* genes were responsive to NAA treatment in shoots and roots, respectively (Fig. 6a). Further, we have also shown that 9 genes in shoots and 10 genes in roots were responsive to SA (Fig. 6b). Auxin responsive elements (AuxREs) and SA responsive elements (SAREs) have been shown to be involved in response to auxin and SA stimuli, respectively [53, 54]. In order to build our knowledge regarding how these genes are induced by NAA and SA, a comprehensive analysis of *GhPIN* promoters was performed. Our results showed that 6 out of 8 highly expressed genes possessed at least one AuxRE in their promoter regions (Fig. 6c). In addition, 4 genes, which were up-regulated in both shoots and roots under SA treatment, contained a predicted SARE in their promoter regions (Fig. 6d).

## Discussion

In this work, 17 *PIN* genes were identified in *G. hirsutum*, including 8 that originated from the At subgenome and 9 from the Dt subgenome (Additional file 2: Table S1). Among them, 5 genes from the A genome and 2 from the D genome were lost in the At and Dt subgenomes, which is consistent with previous results that the allotetraploid genome suffered a higher rate of gene loss relative to the diploid genomes, and that more genes were lost in the At subgenome during polyploidization [34, 35]. Genes in the *PIN5* group were observed in *G. arboreum* and *G. raimondii*, but not found in *G. hirsutum* (Fig. 1), suggesting that these genes evolved from a common ancestor, but were then lost in *G. hirsutum*. Chromosomal distribution of *GhPIN* genes showed that 12 out of the 17 genes are distributed across 8 chromosomes (Fig. 2a).

Genetic analysis in *Arabidopsis* has shed light on the function of several *PIN* genes. Several members of the *PIN* gene family of auxin efflux carriers, such as *AtPIN1*, *AtPIN2*, *AtPIN3*, *AtPIN4*, and *AtPIN7*, are well-known to be involved in cell-to-cell auxin transport in the root [55]. In particular, *AtPIN1* and *AtPIN3* were found to be the major auxin transport facilitators mediating polar auxin re-allocation from the shoot to the root tip [12, 55, 56]. The two *PIN* genes showed high expression in root, thereby determining meristem size and consequently growth rates of the primary root [56, 57]. In this study, we found that both main and lateral roots in *G. hirsutum* were longer than those from its diploid ancestor (Figs. 3a and 4a). qRT-PCR analysis revealed that *GhPIN1–3* and *GhPIN2* were are strongly expressed in both main and lateral roots of *G. hirsutum* (Fig. 4c). The differential and high-level expression of these genes seems to contribute to increased root length in *G. hirsutum*. Lodging, referred to as the permanent displacement of aboveground parts, greatly depends on root length and strength [15]. Lodging is a common phenomenon that causes yield reductions and makes plants difficult to harvest. *GhPIN1–3* and *GhPIN2* are required for cotton root development, which can be further used in breeding programs to selecting genotypes that are lodging-resistance.

Previous results revealed that accumulation of the plant hormone indole 3-acetic acid (IAA) in the epidermis of cotton ovules significantly increased the number of lint fibers, an important component of fiber yield [29]. In *Arabidopsis*, mutation of genes involved in auxin polar transport facilitators resulted in defects in distal organization of roots [39]. Our results showed that *GhPIN1–3* and *GhPIN2* genes might play an important role in regulating both main and lateral root development (Figs. 3 and 4). Auxin may function in both the promotion of both fiber initiation and cotton root growth, implying that auxin may play a dual functional role in cotton development.

Soybean *PIN* genes were previously shown to be induced by various abiotic stresses and plant hormones [19]. *PIN* genes in *Sorghum bicolor* were also found to be differentially up-regulated after phytohormone treatment and under abiotic stress [58]. Additionally, auxin transporter gene families in maize were reported to be responsive to different abiotic stresses [15]. In the present study, several *PIN* genes were shown to be highly expressed in cotton plants grown under drought, salt and dehydration treatments (Fig. 5). Further gene-specific overexpression or analysis of *PIN* knockout plants may be helpful to unravel their functions. 10 out of 17 *PINs* expressed in cotton roots were induced by NAA and SA treatment (Fig. 6a and b). When analyzing the promoter regions of the 10 *PIN* genes upregulated by hormone treatment, 6 and 4 were found to contain AuxREs and SAREs in their promoter regions, respectively (Fig. 6c and d), indicating these elements are very important for *PIN* genes to respond to NAA and SA stimuli. These findings provide clues towards the identification of more candidates with potential roles in phytohormone stimuli.

## Conclusions

Our study provided a comprehensive analysis of the *PIN* gene family in *G. hirsutum*. We showed that *PIN1–3* and *PIN2* are involved in cotton root development. This study will help us to elucidate the precise role of *PIN* genes in cotton root development and in adaption to abiotic stress. Our findings will also further help breeding efforts to develop and select the lodging-resistant varieties in the future.

## Additional files

Additional file 1: Table S4. Primers for qRT-PCR experiments. (PDF 52 kb)

Additional file 2: Table S1. Analysis of *G. hirsutum PIN* genes and their corresponding orthologues in the AA and DD genomes. (PDF 16 kb)

Additional file 3: Table S2. The coding sequences of 17 GhPIN genes identified in *G. hirsutum*. (PDF 31 kb)

Additional file 4: Figure S1. Multiple sequence alignment of the deduced amino acid sequences of predicted PINs from *G. hirsutum*. (PDF 2431 kb)

Additional file 5: Table S3. The number of *PIN* genes identified across 7 plant species. (PDF 89 kb)

Additional file 6: Figure S2. Measurements of lateral root number of three-week-old *G. hirsutum* and *G. arboreum* seedlings. (PDF 100 kb)

## Abbreviations

ARF: Auxin response factor
AuxRE: Auxin-responsive element
CTAB: Hexadecyl trimethyl ammonium Bromide
IAA: indole-3-acetic acid
NAA: 1-Naphthaleneacetic acid
SA: Salicylic acid
UBQ: Ubiquinone
WGD: whole-genome duplication

## References

[1] Benjamins R, Scheres B (2008). Auxin: the looping star in plant development. Annu Rev Plant Biol.

[2] Vanneste S, Friml J (2009). Auxin: a trigger for change in plant development. Cell.

[3] Chapman EJ, Estelle M (2009). Mechanism of auxin-regulated gene expression in plants. Annu Rev Genet.

[4] Robert HS, Grunewald W, Sauer M, Cannoot B, Soriano M, Swarup R, Weijers D, Bennett M, Boutilier K, Friml J (2015). Plant embryogenesis requires AUX/LAX-mediated auxin influx. Development.

[5] Remy E, Cabrito TR, Baster P, Batista RA, Teixeira MC, Friml J, Sa-Correia I, Duque P (2013). A major facilitator superfamily transporter plays a dual role in polar auxin transport and drought stress tolerance in *Arabidopsis*. Plant Cell.

[6] Vieten A, Sauer M, Brewer PB, Friml J (2007). Molecular and cellular aspects of auxin-transport-mediated development. Trends Plant Sci.

[7] Jones AR, Kramer EM, Knox K, Swarup R, Bennett MJ, Lazarus CM, Leyser HM, Grierson CS (2009). Auxin transport through non-hair cells sustains root-hair development. Nat Cell Biol.

[8] Kierzkowski D, Lenhard M, Smith R, Kuhlemeier C (2013). Interaction between meristem tissue layers controls phyllotaxis. Dev Cell.

[9] Swarup R, Kargul J, Marchant A, Zadik D, Rahman A, Mills R, Yemm A, May S, Williams L, Millner P (2004). Structure-function analysis of the presumptive *Arabidopsis* auxin permease AUX1. Plant Cell.

[10] Band LR, Wells DM, Fozard JA, Ghetiu T, French AP, Pound MP, Wilson MH, Yu L, Li W, Hijazi HI (2014). Systems analysis of auxin transport in the *Arabidopsis* root apex. Plant Cell.

[11] Petrasek J, Friml J (2009). Auxin transport routes in plant development. Development.

[12] Adamowski M, Friml J (2015). PIN-dependent auxin transport: action, regulation, and evolution. Plant Cell.

[13] Peret B, Swarup K, Ferguson A, Seth M, Yang Y, Dhondt S, James N, Casimiro I, Perry P, Syed A (2012). AUX/LAX genes encode a family of auxin influx transporters that perform distinct functions during *Arabidopsis* development. Plant Cell.

[14] Barbez E, Kubes M, Rolcik J, Beziat C, Pencik A, Wang B, Rosquete MR, Zhu J, Dobrev PI, Lee Y (2012). A novel putative auxin carrier family regulates intracellular auxin homeostasis in plants. Nature.

[15] Yue R, Tie S, Sun T, Zhang L, Yang Y, Qi J, Yan S, Han X, Wang H, Shen C (2015). Genome-wide identification and expression profiling analysis of ZmPIN, ZmPILS, ZmLAX and ZmABCB auxin transporter gene families in maize *(Zea mays L*.) under various abiotic stresses. PloS one.

[16] Ding Z, Galvan-Ampudia CS, Demarsy E, Langowski L, Kleine-Vehn J, Fan Y, Morita MT, Tasaka M, Fankhauser C, Offringa R (2011). Light-mediated polarization of the PIN3 auxin transporter for the phototropic response in *Arabidopsis*. Nat Cell Biol.

[17] Kleine-Vehn J, Ding Z, Jones AR, Tasaka M, Morita MT, Friml J (2010). Gravity-induced PIN transcytosis for polarization of auxin fluxes in gravity-sensing root. Proc Natl Acad Sci.

[18] Petrášek J, Mravec J, Bouchard R, Blakeslee JJ, Abas M, Seifertová D, Dhonukshe P (2006). PIN proteins perform a rate-limiting function in cellular auxin efflux. Science.

[19] Wang HZ, Yang KZ, Zou JJ, Zhu LL, Xie ZD, Morita MT, Tasaka M, Friml J, Grotewold E, Beeckman T (2015). Transcriptional regulation of *PIN* genes by FOUR LIPS and MYB88 during *Arabidopsis* root gravitropism. Nat Commun.

[20] Ding Z, Wang B, Moreno I, Duplakova N, Simon S, Carraro N, Reemmer J, Pencik A, Chen X, Tejos R (2012). ER-localized auxin transporter PIN8 regulates auxin homeostasis and male gametophyte development in *Arabidopsis*. Nat Commun.

[21] Mravec J, Skupa P, Bailly A, Hoyerova K, Krecek P, Bielach A, Petrasek J, Zhang J, Gaykova V, Stierhof YD (2009). Subcellular homeostasis of phytohormone auxin is mediated by the ER-localized PIN5 transporter. Nature.

[22] Guo X, Qin Q, Yan J, Niu Y, Huang B, Guan L, Li Y, Ren D, Li J, Hou S (2015). TYPE-ONE PROTEIN PHOSPHATASE4 regulates pavement cell interdigitation by modulating PIN-FORMED1 polarity and trafficking in *Arabidopsis*. Plant Physiol.

[23] Alonso-Peral MM, Candela H, Del Pozo JC, Martinez-Laborda A, Ponce MR, Micol JL (2006). The HVE/CAND1 gene is required for the early patterning of leaf venation in *Arabidopsis*. Development.

[24] Luschnig C, Gaxiola RA, Grisafi P, Fink GR (1998). EIR1, a root-specific protein involved in auxin transport, is required for gravitropism in *Arabidopsis thaliana*. Genes Dev.

[25] Utsuno K, Shikanai T, Yamada Y, Hashimoto T (1998). Agr, an Agravitropic locus of *Arabidopsis thaliana*, encodes a novel membrane-protein family member. Plant Cell Physiol.

[26] Müller A, Guan C, Gälweiler L, Tänzler P, Huijser P, Marchant A, Palme K (1998). AtPIN2 denfines a locus of *Arabidopsis* for root gravitropism control. EMBO J.

[27] Wang JR, Hu H, Wang GH, Li J, Chen JY, Wu P (2009). Expression of PIN genes in rice (*Oryza sativa L*.): tissue specificity and regulation by hormones. Mol Plant.

[28] Beasley CA, Egli MA, Chang SR, Radin JW (1979). Independent control of fiber development and nitrate reduction in cultured cotton ovules. Plant physiology.

[29] Zhang M, Zheng X, Song S, Zeng Q, Hou L, Li D, Zhao J, Wei Y, Li X, Luo M (2011). Spatiotemporal manipulation of auxin biosynthesis in cotton ovule epidermal cells enhances fiber yield and quality. Nat Biotechnol.

[30] Zhang M, Xiao Y, Zeng J, Pei Y. PIN-formed protein, a door to reveal the mechanism for auxin-triggered initiation of cotton fiber. Plant Signal Behav. 2017; doi:10.1080/15592324.2017.1319031. 10.1080/15592324.2017.1319031 PMC5501223 28426370

[31] Zhang M, Zeng JY, Long H, Xiao YH, Yan XY, Pei Y. Auxin regulates cotton fiber initiation via GhPIN-mediated auxin transport. Plant and Cell Physiology, 2016: pcw203. doi: 10.1093/pcp/pcw203. 10.1093/pcp/pcw203 28034911

[32] Paterson AH, Wendel JF, Gundlach H, Guo H, Jenkins J, Jin D, Llewellyn D, Showmaker KC, Shu S, Udall J (2012). Repeated polyploidization of *Gossypium* genomes and the evolution of spinnable cotton fibres. Nature.

[33] Wang K, Wang Z, Li F, Ye W, Wang J, Song G, Yue Z, Cong L, Shang H, Zhu S (2012). The draft genome of a diploid cotton *Gossypium raimondii*. Nat Genet.

[34] Zhang T, Hu Y, Jiang W, Fang L, Guan X, Chen J, Zhang J, Saski CA, Scheffler BE, Stelly DM (2015). Sequencing of allotetraploid cotton (*Gossypium hirsutum L*. acc. TM-1) provides a resource for fiber improvement. Nat Biotechnol.

[35] Li F, Fan G, Lu C, Xiao G, Zou C, Kohel RJ, Ma Z, Shang H, Ma X, Wu J (2015). Genome sequence of cultivated upland cotton (*Gossypium hirsutum* TM-1) provides insights into genome evolution. Nat Biotechnol.

[36] Li F, Fan G, Wang K, Sun F, Yuan Y, Song G, Li Q, Ma Z, Lu C, Zou C (2014). Genome sequence of the cultivated cotton *Gossypium arboreum*. Nat Genet.

[37] Liu X, Zhao B, Zheng HJ, Hu Y, Lu G, Yang CQ, Chen JD, Chen JJ, Chen DY, Zhang L (2015). *Gossypium barbadense* Genome sequence provides insight into the evolution of extra-long staple fiber and specialized metabolites. Sci Rep.

[38] Yuan D, Tang Z, Wang M, Gao W, Tu L, Jin X, Chen L, He Y, Zhang L, Zhu L (2015). The genome sequence of Sea-Island cotton (*Gossypium barbadense*) provides insights into the allopolyploidization and development of superior spinnable fibres. Sci Rep.

[39] Finn RD, Coggill P, Eberhardt RY, Eddy SR, Mistry J, Mitchell AL, Potter SC, Punta M, Qureshi M, Sangrador-Vegas A (2016). The Pfam protein families database: towards a more sustainable future. Nucleic Acids Res.

[40] Letunic I, Doerks T, Bork P (2015). SMART: recent updates, new developments and status in 2015. Nucleic Acids Res.

[41] Thompson JD, Gibson TJ, Plewniak F, Jeanmougin F, Higgins DG (1997). The CLUSTAL_X windows interface: flexible strategies for multiple sequence alignment aided by quality analysis tools. Nucleic Acids Res.

[42] Zou C, Lu C, Shang H, Jing X, Cheng H, Zhang Y, Song G (2013). Genome-wide analysis of the sus gene family in cotton. J Integr Plant Biol.

[43] Soderlund C, Bomhoff M, Nelson WM (2011). SyMAP v3.4: a turnkey synteny system with application to plant genomes. Nucleic acids research.

[44] Ji SJ, Lu YC, Feng JX, Wei G, Li J, Shi YH, Fu Q, Liu D, Luo JC, Zhu YX (2003). Isolation and analyses of genes preferentially expressed during early cotton fiber development by subtractive PCR and cDNA array. Nucleic Acids Res.

[45] Chai C, Wang Y, Joshi T, Valliyodan B, Prince S, Michel L, Xu D, Nguyen HT (2015). Soybean transcription factor ORFeome associated with drought resistance: a valuable resource to accelerate research on abiotic stress resistance. BMC Genomics.

[46] Jin X, Li Q, Xiao G, Zhu YX (2013). Using genome-referenced expressed sequence tag assembly to analyze the origin and expression patterns of *Gossypium hirsutum* transcripts. J Integr Plant Biol.

[47] Higo K, Ugawa Y, Iwamoto M, Korenaga T (1999). Plant cis-acting regultory DNA elements (PLACE) database. Nucleic Acids Res.

[48] Lescot M, Déhais P, Thijs G, Marchal K, Moreau Y, Van de Peer Y, Rombauts S (2002). PlantCARE, a database of plant cis-acting regulatory elements and a portal to tools for in silico analysis of promoter sequences. Nucleic Acids Res.

[49] Wang JJ, Guo HS (2015). Cleavage of INDOLE-3-ACETIC ACID INDUCIBLE28 mRNA by microRNA847 upregulates auxin signaling to modulate cell proliferation and lateral organ growth in Arabidopsis. Plant Cell.

[50] Habets ME, Offringa R (2014). PIN-driven polar auxin transport in plant developmental plasticity: a key target for environmental and endogenous signals. The New phytologist.

[51] Zhao Y (2010). Auxin biosynthesis and its role in plant development. Annu Rev Plant Biol.

[52] Shah J (2003). The salicylic acid loop in plant defense. Curr Opin Plant Biol.

[53] Pieterse CM, Van Loon LC (2004). NPR1: the spider in the web of induced resistance signaling pathways. Curr Opin Plant Biol.

[54] Ulmasov T, Hagen G, Guilfoyle TJ (1997). ARF1, a transcription factor that binds to auxin response elements. Science.

[55] Krecek P, Skupa P, Libus J, Naramoto S, Tejos R, Friml J (2009). E. Z. The PIN-FORMED (PIN) protein family of auxin transporters. Genome biology.

[56] Blilou I, Xu J, Wildwater M, Willemsen V, Paponov I, Friml J, Heidstra R, Aida M, Palme K (2005). B S. The PIN auxin efflux facilitator network controls growth and patterning in *Arabidopsis* roots. Nature.

[57] Omelyanchuk NA, Kovrizhnykh VV, Oshchepkova EA, Pasternak T, Palme K, Mironova VV. A detailed expression map of the PIN1 auxin transporter in *Arabidopsis thaliana* root. BMC plant biology. 2016; 16 Suppl 1:5. 10.1186/s12870-015-0685-0 PMC4895256 26821586

[58] Shen C, Bai Y, Wang S, Zhang S, Wu Y, Chen M, Jiang D, Qi Y (2010). Expression profile of PIN, AUX/LAX and PGP auxin transporter gene families in *Sorghum bicolor* under phytohormone and abiotic stress. FEBS J.

