# Supplementary material for: The PIN gene family in cotton (Gossypium hirsutum): genome-wide identification and gene expression analyses during root development and abiotic stress responses
Source: BMC Genomics. 2017 Jul 3;18:507. doi: 10.1186/s12864-017-3901-5 (PMC5496148; doi:10.1186/s12864-017-3901-5)
Supplement: Supplementary file 1 — Primers for qRT-PCR experiments. (PDF 52 kb) [file 12864_2017_3901_MOESM1_ESM.pdf]

Table S4. Primers for qRT-PCR experiments.

Gene ID	Sense primer sequence	Antisense primer sequence
GhPIN1-2-D	ACCCATTATCCGGTTCCTAA	TATGCCCTTCAACTTTCCCT
GhPIN1-3-D	ATGTTTACGGTTTGTCTGCC	GTCGGATACAGGAGAAGCAC
GhPIN1-4-A	GTAATAAGGCAGCAACGAAC	TGAATAAGCCAAGGCTAAAC
GhPIN6-D	GGGAGCAAACAAGAAATGCC	ACTGAACATTGCCATCCCAA
GhPIN8-2-A	TGTTGGCTATGGGTCTCACT	CGATAACGAAACGTCTCCTC
GhPIN8-2-D	AGTATTCGCCTTCGTTGTTT	GCTTCCGTGTCCTCTATGTC
GhPIN1-1-D	GTGTTACCGACCAGAAAGAA	AAGCCTTGTCATTACACTCG
GhPIN2-D	TATTATCCGATGCTGGTCTG	GTGCTAAGTATGTCGGGATG
GhPIN8-1-D	CAACCCTTACAAGATGAACC	GTAGAACGACAATCTGAGCC
GhPIN2-A	TTTTCCTATGGCAAGCCTTCA	CGTATATGTCCTTTCCAGTGATCAT
GhPIN1-4-D	CCGTAATACTTTCCGTGATG	TGTTGCCAATACCACTCCTA
GhPIN8-1-A	CAACCCTTACAAGATGAACC	GTAGAACGACAATCTGAGCC
GhPIN9-A	GGTTGAGAGTGTGAAGCAAG	CATCCCAAGACCTGCACT
GhPIN3-D	GGTGGGTATTGGTTGTGATAG	AAGATAACCGCAGTGCTAAGA
GhPIN6-A	GGGAGCAAACAAGAAATGCC	CTGAACATTGCCATCCCAAG
GhPIN1-3-A	ATGTTTACGGTTTGTCTGCC	GGGAGACAGCCACTCTAACC
GhPIN3-A	ATCCGTCACGAAGAACGCTAA	TGCTCAGACCGTCCAGATTG
GaPIN1-2	GTGTTACCGACCAGAAAGAA	AAGCCTTGTCATTACACTCG
GaPIN1-3	AGGGAAAGTTGAAGGGCATAG	CACCTGTTGGAGGCATTGTA
GaPIN1-1	TATGGAGCAAACGAGCAGAA	TTCGTGGTTGTTGGTCATTT
GaPIN3	CGGAATCCCAACACTTACTC	AGTCCCAATAGAATGTAGTAAACC
GaPIN5-1	ACCGCCTCGTCTGCTATTTC	TCTCCTGTTCTCCGCCATCT
GaPIN6	CATCCTCCATTTGCCTCACC	GCATCCGACGAAGCATACCC
GaPIN8	TATCGGTGAAATGGTGGAAG	GCAAGAAATCGGCAAGTATG
GaPIN2	ACTCTTCTAAACCTACCCATCA	TATCTCCAAATCCTTGTCCC
GaPIN6-1	TGATTCAAGTTGTGGTGCTG	TGGTTAGATGATGTTGTGGC
GaPIN5-2	AACCGCCTCGTCTGCTATTT	TCTCCTGTTCTCCGCCATCT
GaPIN4	AATCCCAATACATATTCCAGTC	AAAGATAACCGCAGTGCTAA
UBQ7	GGCATTCCACCTGACCAACAA	CCGCATTAGGGCACTCTTTTC
