# Supplementary material for: The PIN gene family in cotton (Gossypium hirsutum): genome-wide identification and gene expression analyses during root development and abiotic stress responses
Source: BMC Genomics. 2017 Jul 3;18:507. doi: 10.1186/s12864-017-3901-5 (PMC5496148; doi:10.1186/s12864-017-3901-5)
Supplement: Supplementary file 2 — Analysis of G. hirsutum PIN genes and their corresponding orthologues in the AA and DD genomes. (PDF 16 kb) [file 12864_2017_3901_MOESM2_ESM.pdf]

Table S1. Analysis of *G. hirsutum PIN* genes and their corresponding orthologues in AA and DD genomes.

Gene ID	Number of intron	Sub-genome	Orthologous	Diploid cotton	Name
CotAD_03159	5	At	Cotton_A_13051	*G. arboreum*	PIN1-3-A
CotAD_68019	5	At	Cotton_A_31611	*G. arboreum*	PIN1-4-A
CotAD_23365	6	At	Cotton_A_29759	*G. arboreum*	PIN2-A
CotAD_13788	5	At	Cotton_A_40424	*G. arboreum*	PIN3-A
CotAD_05841	5	At	Cotton_A_19614	*G. arboreum*	PIN6-A
CotAD_40873	5	At	Cotton_A_29004	*G. arboreum*	PIN8-1-A
CotAD_46231	2	At	Cotton_A_16465	*G. arboreum*	PIN8-2-A
CotAD_58548	4	At	Cotton_A_30421	*G. arboreum*	PIN9-A
CotAD_21821	4	Dt	Gorai.008G290000.1	*G. raimondii*	PIN1-1-D
CotAD_02796	5	Dt	Gorai.007G003800.1	*G. raimondii*	PIN1-2-D
CotAD_70220	5	Dt	Gorai.006G191800.1	*G. raimondii*	PIN1-3-D
CotAD_04916	1	Dt	Gorai.007G352200.1	*G. raimondii*	PIN1-4-D
CotAD_43404	6	Dt	Gorai.009G001600.1	*G. raimondii*	PIN2-D
CotAD_74791	5	Dt	Gorai.002G179000.1	*G. raimondii*	PIN3-D
CotAD_69056	6	Dt	Gorai.005G082500.1	*G. raimondii*	PIN6-D
CotAD_47634	5	Dt	Gorai.012G056200.1	*G. raimondii*	PIN8-1-D
CotAD_15657	3	Dt	Gorai.011G096500.1	*G. raimondii*	PIN8-2-D
