# Supplementary material for: The PIN gene family in cotton (Gossypium hirsutum): genome-wide identification and gene expression analyses during root development and abiotic stress responses
Source: BMC Genomics. 2017 Jul 3;18:507. doi: 10.1186/s12864-017-3901-5 (PMC5496148; doi:10.1186/s12864-017-3901-5)
Supplement: Supplementary file 3 — The coding sequences of 17 GhPIN genes identified in G. hirsutum. (PDF 31 kb) [file 12864_2017_3901_MOESM3_ESM.pdf]

Table S2. The coding sequences of 17 *GhPIN* genes identified in *G. hirsutum*.

>GhPIN1-2-D
ATGATCTCCATTACAGATTTCTACCATGTCATGACTGCCATGGTTCCACTTTACGTGGCCATGGTTTTAGC
TTATGGCTCGGTGAAATGGTGGAAGATTTTCACTCCTGATCAGTGTTCAGGCATCAACCGTTTTGTTGCTC
TCTTTGCTGTCCCTCTCCTTTCTTTTCACTTCATCGCTTCTAATGACCCTTATTCTATGAACTTTCGTTTCAT
AGCTGCTGATACACTTCAAAAACTTATAGTTCTTGCAGTACTTGCTGTTTGGACCAAGGTCAGTAAAAGAG
GTTGTTTGGAATGGACCATTACCCTTTTTTCACTTTCTACTCTTCCAAACACTTTGGTTATGGGTATTCCTT
TACTTAAAGGAATGTATGGGGATTTTTCAGGGAGTTTAATGGTGCAAATAGTTGTTCTTCAATGTATTATTT
GGTACACTTTGATGCTTTTCTTGTTTGAATATAGAGCGGCTAAAATGCTTATCTCTGAGCAATTTCCCGGC
ACAGCAGGCTCTATTGTTTCTATCCATGTTGATTCAGATGTCGTGTCGCTTGATGGTCGACAACCTATTGA
AACCGAAGCTGAGATTAAAGAAGATGGTAAGCTTCATATTACTGTCAGAAAATCCAATGCTTCAAGATCAG
ATATTTTCTCAAGAAGGTCCCATGGTTTTACATCTACAACTCCACGTCCTTCGAATCTAACCAATGCTGAG
ATATACTCTTTGCAATCATCGAGGAACCCAACACCAAGAGGCTCGAGCTTTAACCACACTGATTTTTATTC
CATGATGGCTGGGGGACGCAATTCAAATTTCGGTTCTGCAGATGTTTACGGTTTGTCTGCTTCGCGTGGA
CCAACTCCGAGACCATCCAACTATGAAGAGGATGGTGCAGCCGCTGGTAAACCGAGGTTCCATTACCAA
GCACCAGGCGGTGGTGGCGGTGGTGGCGGTGCAACCCATTATCCGGTTCCTAATCCTGGTATGTTTTCG
CCGACTGGTTCTAAACCCCTCGGTGGTGGTAATGCAAATGCAAATGCAAAGAGGCCTAATGGTCATCCC
CAACAAAAGTCTGAAGATGGCGGTAGGGATCTTCCAGTGTTTGTTTGGAGTTCTAGCGCTTCTCCTGTTT
CCGATGTCTTTGGCGGTACCGGCCATGATTACGGCGCTGCCGACCAGAAAGATGTTAGATTGGCTGTCT
CCCCAGGGAAAGTTGAAGGGCATAGAGAGAACCGTGAAGAGTACATGGAAAGAGAAGACTTGAGCTTCG
CTAAAGGAGGAATGAACGGAGAAATGAACAAACACGAAGGTGACAAGGTGGGGGATGGCAATGGCAAG
CCCAATACAATGCCTCCGACAAGTGTAATGACAAGGCTGATACTGATCATGGTCTGGAGAAAGCTTATAA
GGAATCCCAACACCTATTCAAGCTTGATAGGTCTCACTTGGTCTTTAATCTCGTTCAGGTGGAATGTAGAA
ATGCCAGCGATTATAGCAAAGTCCATTTCAATTCTGTCAGATGCAGGACTTGGCATGGCCATGTTCAGTC
TCGGTCTGTTCATGGCATTGCAACCAAGGATCATAGCATGTGGGAATTCGGTTGCAGCTTTTGCAATGGC
CGTGAGATTCCTTACAGGTCCAGCTGTCATGGCAGCCGCTTCCATTGCTGTTGGTCTCCGTGGCGTTCTC
TTACGGGTTGCCATTGTCCAGGCAGCTCTTCCTCAAGGAATAGTACCCTTTGTCTTTGCAAAGGAATACA
GCCTGCACCCTGATATTCTCAGCACAGCTGTAATCTTTGGGATGCTAATAGCTTTGCCCATAACACTTGTC
TACTATATATTATTGGGGATATAA

>GhPIN1-3-A
ATGATCACTTTAATTGACTTCTACCATGTAATGACTGCAATGGTGCCACTGTACGTGGCCATGATTTTAGC
TTACGGCTCAGTGAAATGGTGGAAGATCTTCTCTCCTGGTCAGTGTTCGGGGATTAATCGCTTTGTTGCT
CTCTTTGCCGTCCCTCTCCTCTCTTTCCACTTCATCGCCTCTAACGATCCTTACGCTATGAACTTTCGTTT
CATAGCAGCTGATACCCTCCAAAAAGTTATGGTTCTTGGAATACTGGCAGTTTGGTCCAAGGTGAGCAAA
AGGGGTTGCTTGGAATGGACCATCACCCTATTTTCACTCTCTACTCTTCCCAACACTTTGGTGATGGGCA
TCCCTTTGCTGAAAGGAATGTACGGGGAGTTTTCAGGAAGCTTAATGGTGCAGATAGTTGTCCTTCAATG
CATTATTTGGTACACTTTGATGCTTTTCATGTTTGAATATAGAGGTGCCAAAATGCTGATATCTGAGCAATT
CCCCGACACTGCTGGCTCTATTGTTTCAATCCATGTAGACTCAGATATCATGTCACTCGATGGTCGACAA
CCTCTAGAAACTGAAGCTGAGATCAAAGAAGATGGTAAGCTCCATGTTACTGTCAGGAAATCCAATGCCT
CAAGATCAGATATTTTCTCAAGAAGGTCCCAGGGTTTATCTTCAACAACTCCACGCCCTTCCAATCTGACC
AATGCTGAGATCTACTCGTTGCAATCATCAAGAAACCCAACACCAAGAGGCTCAAGCTTTAATCACACTG
ATTTCTACTCTATGATGGCTGGGGGACGCAACTCCAATTTCGGTGCTGCAGATGTTTACGGTTTGTCTGC
CTCTCGAGGACCAACTCCCAGACCATCCAATTATGAGGAGGATGGTACAGGCATGGGTAAACCAAGGTT
CCATTACCACGCACAAGGTGGCGCAGGTGCAGCCCATTATCCGGCTCCTAATCCGGGTATGTTCTCTCC
CAATGGGTCTAAAGCTAATACAAAGAAGCCTAACGATCAGGCTCAGCAAAAGGCTGAAGATGGTGGTAG
GGATCTCCATATGTTTGTTTGGAGTTCAAGTGCTTCTCCTGTATCCGACGTCTTTGGTGGTGGTGGTGGC
CATGAATATGGAGCAAACGAGCAGAAAGAGGTTAGAGTGGCTGTCTCCCCAGGGAAAGCGGAAGGACAT
AGAGAGAATAACGAGGAGTACATGGAGAGAGAGGATTTCAGCTTTGGGAACCGAGGATTGGAACGAGAA
ATGACCAACAACCACGAAGGTGACAAAGTGGGGGATGGCAAGCCCAAAACTATGCCTCCTGCAAGTGTC
ATCACAAGGCTGATACTGATCATGGTTTGGAGAAAGCTTATCAGAAATCCCAACACCTATTCAAGCTTAAT
AGGACTCACTTGGTCTCTAATCTCATTCAGGTGGAATGTACAAATGCCTGCCATAATAGCCAAGTCCATTT
CCATACTGTCAGATGCAGGGCTTGGCATGGCCATGTTCAGTCTTGGTCTGTTCATGGCATTGCAACCGAG
GATCATAGCATGTGGAAATTCCGTTGCAGCTTTTGCCATGGGTGTGAGATTCCTTGCAGGTCCAGCTGTG
ATGGCAGCCGCTTCCATAGCTGTCGGACTCCGTGGCGTTCTCTTACACGTTGCCATTGTGCAGGCAGCT
CTCCCACAAGGCATTGTCCCCTTCGTCTTTGCCAAGGAATACAACCTACACCCTGATATTCTCAGCACTG
CTGTTATTTTTGGAATGCTAATAGCCTTGCCCATAACGCTTGTCTACTACATTTTATTGGGATTATGA

>GhPIN1-4-D
ATGATCAGTGCAACAGACCTTTACCATGTCCTCACCGCCGTTGTACCTCTCTACGTAGCCATGATTTTAGC
TTACGGTTCAGTCAAATGGTGGAAAATCTTCACCCCCGATCAATGTTCCGGCATCAACCGTTTCGTCGCT
TTATTTGCCGTCCCTTTACTTTCATTCCATTTCATATCAACTAACAATCCATACACCATGAACTTGAAGTTC
ATAGCCGCTGATACCCTTCAAAAGCTCATCGTCCTAGTACTTTTAGCCATATGGTCAAGAACTAGCTCAAG
GGGTTCCTTAGAATGGTCCATAACCTTATTTTCACTCTCAACACTCCCCAATACTTTAGTAATGGGCATCC
CTTTGTTAAAAGGCATGTATGGGGATTATTCAGGGAGTTTAATGGTTCAAATAGTGGTTTTACAATGTATA
ATTTGGTACACTTTGATGTTATTCTTGTTTGAATATCGTGGTGCTAAGCTATTAATAGCTGAACAATTCCCA
GATACTGCTGGTTCTATAATTTCATTTAAAGTTGATTCTGATGTTTTGTCATTAGATGGTAAAGAACCTTTA
CAAACCGATGCTGAAGTCGGTGATGATGGGAAACTTCATGTTACTGTAAGGAAATCAACTAGCTCAAGAT
CGGAAATCTTTTCTCGCCGGTCACATGGTTTGAATTCCGGTATGGGGTTAACACCAAGACCTTCTAATTTA
ACCAATGCTGAAATTTATTCACTTCAATCATCTAGGAATCCAACACCAAGAGGTTCTAGTTTTAACCATACT
GATTTTTATTCAATGGTGAACGGGGGGAAGAACAATGTTAGTCCAAGGCAATCAAATTATGGGATAGGAT
TTGATGAAGAGAATGGCGTTGGGATGTTTGGTAATAATGGTATAAGATCAAATGGTAGTTCATATCCTGCT
CCGACAAGTGCTGGAATCTTTTCTCCGGTGACTGCTCCCAAGAAGGCAAACGGTGGCGGTGACGGTGGT
GGAAAGGATTTGCATATGTTTGTTTGGAGTTCAAGTGCTTCCCCTGTTTCTGAAGGTGGTATACATGTTTT
CAAAGGTGGAGATTATGGCAATGATCATCATCATAAAGATTATGATGAATATGGTAGAGACGAATTCACTT
TCGGTAATAAGGCAGCAACGAACGGTGGTGCCGATCGAGAAGGACCGGTTCTTTCCAAGCTTGGTTCAA
GTTCCACGACTGAACTTAACCCGAAGATTGAAACAAAGCCGGCCGCTATGCCTCCGGCTAGTGTTATGAC
TAGACTTATATTGATCATGGTGTGGCGAAAACTAATCCGGAATCCCAACACTTACTCGAGTTTGATCGGTC
TCACTTGGTCTCTTGTTTCGTTCAAGTGGAATATCCAAATGCCTGCTATAGTAGCCGGTTCGATATCGATT
CTTTCCAATGCAGGTCTCGGAATGGCCATGTTTAGCCTTGGCTTATTCATGGCACTTCAACCAAGGATAAT
TGCATGCGGGAACACGATCGCGACCTTTGCCATGGCGGTTCGGTTCCTAACCGGTCCTGCGGTTATGGC
AGCTGCCTCGATCGCTGTTGGGTTAAGAGGTGTTCTTCTCCGCATTGCTATCGTGCAGGCAGCTCTTCCA
CAAGGGATCGTACCTTTCGTCTTTGCAAAGGAGTACAATGTTCATCCTGATATATTGAGCACAGGGGTTAT
ATTTGGAATGCTGATTGCCCTTCCCATAACACTGGTTTACTACATTCTATTGGGACTTTGA

>GhPIN6-A
ATGATAACAGGGGGTGATTTTTACAAGGTGATGTGTGCGATGGTTCCACTGTATTTCGCAATGATAATAG
CGTACGGATCGGTGAAATGGTGGCGAATATTCAGCCCGGAGCAGTGTTCGGGGATCAACCGGTTCGTG
GCGGTTTTCGCGGTGCCGGTGTTGTCATTCCATTTCATAGCTCAAAACAACCCGTACCAGATGGACACCA
AGTTCATCATAGCGGACACGGTGTCCAAGGTGTTGGTCCTTGCTTTGCTTTCGGTTTGGGCTATCTTCTT
CCCTGGTGGCTCACTCGATTGGCTCATCACCCTTTTCTCCCTCGCCACTTTGCCCAACACTCTCGTCATG
GGCATCCCCTTGCTTAATGCTATGTATGGAGATTTCACTCAAAGTCTCATGGTTCAACTCGTTGTTCTTCA
ATGCATCATCTGGTACACACTATTGCTATTCCTCTTCGAATACAGGGCGGCAACGCTCTTAATCAAAACCC
AGTTCCCGGGCCCCACAGCTGCCACCATTTCCAAATTCGAGCTCGACAACGACGTCATTTCATTGGACG
GTCGTGATCCTCTCCGTACAGAATCCGAAACCGACATCAACGGCCGTATTCGAGTCCGAATCAGGCGGT
CCACGTCATCGGCTCCGGAATCGGCTCTATCATCCTCCATTTGCCTCACCCCTAGAGCATCCAACCTCTC
TAACGCCGACATATTTTCCGTTAACACCCCCGGCGGACCCAACAATAACGAGATTGTATTCTGTAACGGC
GACATGGGGTTCGGTTACCGTGCCGTTAGCCCCCGTTTGTCCGGGTATGCTTCGTCGGATGCTTACTCT
TTGCAGCCGACCCCAAGGGCTTCTAATTTTAATGAAATGGATGTGATTACTACGGCAGCCGGGAATACAC
CAATTTGGATGAGGTCTCCCGTGGCTGGTGGGAAAGTTTTCCGGCAGCCGTCTCCGGTGGTTCCTCCTA
CAAAAATGGTTTGGGACTGTCAAGATGGTGGTGGGGATGATAATAGGCAGGGCTTCAAAGACCTTGGGG
AGAAGGAAATTAGCTTTAGAGACAACACCAAAATAACAGTGGCGGAGATGAATGGAGATGGGAAGGAAG
GTGAAGTGGGGAGCAAACAAGAAATGCCAAAGGCCATAGTGATGGTGAGACTCATTTTAATTGTTGTTGG
AAGGAAGCTTTCTCGAAACCCTAATACTTACTCAAGCATCTTAGGGCTTCTTTGGTCTCTCATCTCTTTCA
AGTGGAATGTAGGGATGCCGAGTTTGGTCAAGTATTCAATAAAAATAATCTCCGATGCTGGCCTTGGGAT
GGCAATGTTCAGTTTAGGGTTATTTATGGCACTTCAGCCAAGGATTATTGCATGTGGAACAAAAAGAGCA
ACAATGGGGATGGTGATTCGTTTCCTGTGTGGCCCTGTAATAATGTCCACTGTATCGATAGCTCTGGGAT
TAAGAGGGGCAAAACTACATGCTGCCATCGTACAGGCAGCCCTTCCCCAAGGTATCGTACCATTCGTCTT
TGCAAGAGAATACGGTTTACATCCCGACATATTAAGTACTGGGTAA

>GhPIN3-A
ATGTTATCCACTGCTGCTAGCTTGTACCCTTTGTATGTGACTGTTGGTGGCATTGTGGCTTGTTTTAAACC
ATCTGCTTTTGCTTGGTTCGTGGAGAGAGGACCTTCTTCATATAGCTTCTCGCTTGGGTTGATTATGTTGG
CTATGGGTCTCACTTTAGAACTCAAGGACTTGCTCAATTTGTTCACCCAAAGACCTCTTTCTATTCTATTTG
GATGTGTTGCTCAGTACACAATTATGCCAACTTTTGCTATGATTATTAGCAAAACTTTGGGGCTTTCACCA
TCTCTTTCTGTTGGTTTGATTTTGCTAGGTTGTTGTCCTGGTGGTGCTGCATCCACTGTGGTGACCTTTAT
TGCACGAGGAGACGTTTCGTTATCGACGGTGATGACGGTTTGCACTACTCTTGGTGCAGTGATCCTTACA
CCTCTTTTGACTATGGTTCTAGCAGGAACATATGTTTCTATTGATGCTATTGGACTTTCCATCAGCACCCT

GCAGGTGGTAGTGGCTCCGGTTTTGATCGGTTCTTACCTACAAAGCACATTTCCTTTGGTGGTGAAAATG
ATTACACCATTTGCTCCTTTATTTGCCGTTTTACTTTCATCGCTACTTGCATGCAGTGTTTTCTCGGGAAAT
GTCGTCCGTTTTAATTCCTCAATAGGTAATGCATCATTGGTTTCCGATGCCTCTTTAGTCTCACGCCTCCA
ATCATTATTATCTGGAGGCGTCGGTGCCGTAATACTTTCCGTGATGTTGCTACATTTTACTGGTTTCTTTG
TAGGGTACATATCAGCAACCATTTGTAGATTCCGAGAAGCAGAGCGGCGAGCAATATCAATCGAGGTTG
GCATGCAAAATTCTTCATTAGGAGTGGTGTTGGCAACAACTCATTTCACTTCTCCAGTTGTAGCGTTACCG
CCTGCCATGTCGGCAGTGATTATGAATATAATGGGTAGCAGCTTAGGCTTCTTTTGGAGACAGATCAGTG
GTTCAAAACAGGAACTTGAAGATCAAGAGTGA

>GhPIN8-2-D
ATGAACATCAAACTCGTACTTGCCGACTTTCTTCAGAAAGTATTCGCCTTCGTTGTTTTGATTGCCGTTGG
ACAACTCCGATCTCGGGGCGGTGAGCCGTCTATCATAACTGGCTTCTCGTTATCGACCTTGCCTAACACT
TTGATCTTGGGGATACCGTTGTTGAAGGCCATGTATGGTGACGAACCGGCCGGATTACTGGCTCAGATT
GTTGTTCTTCAAAGCTTGATTTGGTATAATTTACTGTTGCTCATGTTCGAGTTCAATGCTGCAAAGCCAGC
CTCTGAGATTACATCAACTTCAGAAGACATAGAGGACACGGAAGCACAAGGGAAAGAGAGAGAAGAAGA
GGCAGAAACCAGAGCAAGCAAATTCAAGATTATGCTCATTTTCTTGACTGTGGAAAAGAAGCTGGTGGCA
AATCCCAACACTCATGCAGCTTGGCTGGGTCTCATTTGGGCTGGCATTCGATTCAGGTGGGAAATAAAAT
TCCCTGCCATTATTCAGAATTCATTATCAATATTAGCTAGCGGAGGACTTGGTCTATTTATGGCATCACAG
CGTAGCAGAATAGCATGTGGTATACGAATGACAGCAGTAGCCATGGTCATGAAATTCATGGCTGGTCCAG
CTCTAATTGCAGCATCCTCAGCTGCCTTGGGACTTCGGGCAGATTATTAA

>GhPIN1-1-D
ATGATCACTTTAACAGATTTTTACCATGTTATGACCGCAATGGTACCACTTTACGTGGCCATGATTTTAGCT
TACGGTTCAGTGAAATGGTGGAAAATCTTCACACCCGACCAATGTTCCGGCATCAACCGGTTTGTTGCTC
TTTTCGCCGTTCCATTACTTTCATTTCATTTCATAGCTTCAAACAATCCGTACACCATGAACTTCCGGTTCA
TAGCAGCTGATACACTACAAAAGATTATAGTTTTAGTCGTGTTAGCCATTTGGTCCAAAGTAAGTAAAAGG
GGTTGTTTAGAATGGACCATAACATTATTTTCACTTTCAACATTACCCAATACTTTAGTTATGGGTATACCT
TTACTTAAAGGTATGTATGGTGAATTTTCAGGGAGTTTAATGGTACAAATCGTTGTACTTCAATGTATCATT
TGGTATACTTTAATGCTATTTATGTTTGAATATCGAGGTGCTAAGATGTTAATCTCTGAACAATTCCCCGAC
ACTGCTGGTTCTATTGTGTCTATCCATGTCGATTCCGACATCATGTCGCTCGACGGTCGGCAGCCGTTAG
AAACCGAAGCTGAGATTAAAGACGACGGTAAGTTGCATGTTACGGTCCGAAAATCTAATGCTTCGAGGTC
TGATATTTTTTCGAGAAGGTCACAAGGTTTGTCTTCGACTACCCCCCGGCCTTCGAATTTAACGAATGCTG
AGATTTACTCTTTACAATCATCGAGGAATCCAACACCGAGAGGTTCGAGTTTTAACCATACTGATTTTTATT
CTATGATGGCCGGTGGTCGGAACTCGAACTTTGGTTCCGGTGATGTTTATGGTATGTCTGCTTCACGTGG
ACCGACACCCAGACCATCAAATTACGAGGAAGATGGTTCTGGTAAGCCGAGGTTCCATTACCATGGACAA
AGTGGTGTAACGGGACACTACCCGGCTCCTAATCCGGGTATGTTTTCACCAACTGGGTCTAAAGGTGTTA
AGAAACCTAATGGACAAGCTCATCAAAAGGTTGAAGATGGTGGTGGTAAGGATCTTCATATGTTTGTTTG
GAGTTCAAGTGCTTCTCCGGTATCAGATGTGTTCGGCGGCGGCTATGAATATGGTGTTACCGACCAGAAA
GAAGTTCGGTTAGCTGTCTCCCCTGGCAAAGTGGAAGGACATAGAGATAATCAAGAGGAGTTTATGGAAA
GAGATGAGTTCAGTTTTGGGAACAGAAGAGAAATGAACAATGTCCAAGAAAGTGGTGAAAACAACAAAGT
TGGGGAAGCCATTGCCATTGCTAACCCTAAAACCATGCCACCAACGAGTGTAATGACAAGGCTTATATTG
ATTATGGTTTGGAGAAAACTTATTAGAAACCCCAATACTTATTCAAGCTTAATTGGTCTCACTTGGTCATTA
ATCTCATTCAGGTGGAATGTGGAAATGCCTGCAATAATAGCAAAGTCGATATCTATACTGTCAGATGCAG
GTCTCGGCATGGCTATGTTCAGTCTTGGTCTGTTTATGGCATTGCAACCAAAGATCATAGCATGTGGGAA
TTCGGTTGCAGCTTTTGCAATGGCCGTGAGATTCCTTACAGGACCAGCTGTCATGGCTGCTGCTTCCATT
GCTGTTGGTCTTAAAGGCGTTCTCTTACACGTTGCCATTGTCCAGGCAGCTCTCCCACAAGGAATTGTCC
CTTTTGTCTTTGCCAAGGAATACAACGTACACCCTGATATTCTCAGCACAGCGTAA

>GhPIN2-A
ATGATCACTGGAAAGGACATATACGACGTTCTGGCAGCTATTGTGCCCCTATATATTGCTATGATGTTAGC
ATATGGGTCGGTGAGGTGGTGGAAAATATTTACCCCAGATCAATGTTCAGGCATCAACCGTTTTGTTGCA
GTGTTTGCAGTTCCGTTACTCTCCTTCCATTTCATCTCCTCAAATGATCCATATGCTATGAACTACCACTTC
ATAGCTGCCGATTCTCTCCAGAAGGTAGTCATCCTCGTTGCCCTTTTCCTATGGCAAGCCTTCACCAAAC
ATGGCAATCTGGAGTGGATGATTACTCTCTTCTCTCTATCCACTCTCCCCAACACTCTTGTCATGGGGATC
CCCCTTTTAAAGGCTATGTACGGGGATTTCTCCGGCAGCCTTATGGTTCAAATTGTAGTTTTGCAGAGTGT
GATTTGGTACACTCTCATGCTTTTCATGTTTGAATATAGAGGAGCAAAGCTTCTGATCAACGACCAGTTCC
CGGAGACTGCTGGATCTATTACCTCTTTCCGTGTAGAGTCGGATGTTGTTTCACTTAATGGCCGCGAGCC
TCTACAAGCTGACGCTGAGATTGGTGATGACGGAAAACTCCACGTTGTGGTGAGAAGATCAAGTGCGTC
ATCAATGGTATCGTCCTTTAGCAAATCACATGGACTGAATTCCTTAACGTCCATGACTCCCAGAGCCTCAA
ACCTTACCGGGGTAGAAATATACTCTGTCCAGTCATCCAGAGAGCCCACTCCCAGGGCTTCAAGCTTTAA
TCAAAACGATTTCTATGCCATGTTTGCAAGCAAGGCTCCAAGTCCCAAGCATGGCTACACCAACAGCTTC

CAGGGGGCAGTTGGTGATGTTTTTTCGCTGCAATCTTCCAAAGGAGCAACACCAAGAACTTCAAACTTCG
ATGAAGAGATGCTCAAGGTTGCCAAGAGAAGGGGGGGAAGGAGCATGAGCGGCGAGTTATATAACGGT
GGTGGTGGTGGTGGTGTTCCTTCTTATCCACCGCCCAATCCAATTTTTGCGGGGTCAACTAGCGGTGGC
TCTAAGAAGAAGGAAAGTGGCTCAATGCCTAACAAAGAGCTTCACATGGGGGGAAGGAGCATGAGCGGC
GAGTTGTATAACGGTGGTGGTGTTCCTTCTTATCCACCACCCAATCCAATTTTTGCGGGGTCAACTAGCG
GTGGCTCTAAGAAGAAGGAAATTGGCGCAATGCCTAACAAGGAGCTTCACATGTTTGTTTGGAGCTCTAG
TGCTTCTCCTGTGTCGGAAGGGAATCTAAGGCATGCAGTTAATAGAGCTGCCTCTACTGACTTTGACTCT
TCTAAACCTACCCATCAACAAGAAAACGCTGCTTCAAGAGCAATGCATGAGCTAATTGAAAATATGGGTAA
AAGCAGTGGGGGGGACAAGGATTTGGAGATATTAGAGGAGGGATCCAAGTTTCCAACAGGTGGATCTCC
CTTGAGTTGTCAGAAAAAGTTGAACATGGAAGAAGGCGAAGTGGCAAAGAAACAGCAGATGCCTCCTGC
AAGTGTGATGACAAGGCTTATTCTTATCATGGTTTGGAGGAAGCTCATTAGGAACCCTAACACATATTCCA
GTCTTTTCGGCTTGACTTGGTCCCTCATATCTTTCAGGTGGAACATTCAGATGCCAACAATTGTAAGTGGG
TCGATAGCAATATTATCCGATGCTGGTCTGGGAATGGCAATGTTTAGTCTAGGCTTATTCATGGCATTACA
ACCCAGGATCATAGCTTGCGGAAAATCTGTAGCAACATTTTCCATGGCTGTCAGGTTTTTGACGGGTCCC
GCTGTCATTGCTGCAACCTCCATAGCCATTGGTCTTCGTGGGGTTCTTTTGCATGTCGCTATCGTTCAGG
CTGCCCTTCCACAAGGAATAGTTCCATTTGTGTTTGCCAAGGAGTATAATGTTCATCCCGACATACTTAGC
ACTGCGGTTATTTTCGGAATGTTGATTGCTCTGCCCATTACAATACTCTACTATGTGCTTCTTGGACTGTA
A

>GhPIN8-1-A
ATGATTTCCCTGGCAGATGGTTATCATGTTATAGCAGCCACCGTGCCTTTATACTTTGCCATGATTTTAGC
CTACGTATCGGTGAAATGGTGGAAGCTGTTCACACCGGAACAATGCGCAGGCATCAACAAGTTCGTGGC
TAAATTCTCAATCCCACTCTTGTCTTTCCAAGTCATCTCCGAAAACAACCCTTACAAGATGAACCTCAAACT
CATACTTGCCGATTTCTTGCAAAAACTATTGGCTTTCCTTGTCTTGTTTGCCCTCACTAAGCTCAGTTCCC
GGGGTGGTTTATCGTCCATCATAACCGGTCTCTCTTTATCCACCATGCCTAACACTTTAATCCTCGGAATC
CCACTGTTGAGGGCTATGTATGGAGATAAATCAGCCACTCTTTTGGCTCAGATTGTCGTTCTACAAAGCTT
AATTTGGTATAATTTACTCTTGTTTTTGTTTGAGCTAAACGCTACCAAAGCTGCCTCTGAGACCACTGTTG
CAGTTTCACAAGCCTCAGGGGGCGAAGAGGCCCCCGAAGAAGCACAAGGGAAAGGGGGAGGAGAAGA
GACACAAACGAGAGCAAGGAAATCCAAGACAATGCTGATTTTCTTGACAGTGGGGAAGAAGCTAATGGC
GAATCCCAATACCCATGCTACCTTACTTGGTCTTATTTGGGCAAGCATTCAATTCAGGTGGAACATTAAAT
TCCCCGCCATTGTTGAAAAATCAATAGCAATATTATCCAGTGGAGGACTTGGAATGGCAATGTTCAGCTTA
GGTCTATTCATGGCATCACGGCCAAGCATAATAGCTTGTGGTATACGAATGGCGACAGTAGCCATGATCA
TGAAATTCATTGCTGGTCCAGCTCTAATGGCGGCCGCCTCCACCACTCTCGGACTTAAAGGCAAATTATT
GAGAGTGGCAATTGTACAGGCTGCTCTCCCTCAAGGAATCGTTCCATTTGTTTTCGCCAAAGAGTACAAT
GTTCATCCTGATATATTAAGCACAGGGGTAATCTTCGGTATGCTTATTGCCTTACCTGTAGCATTGGTCTA
CTATCTTCTTTTAGCATTGTGA

>GhPIN2-D
ATGATCACTGGAAAGGACATATACGACGTTCTGGCAGCTATTGTGCCCCTATATATTGCTATGATGTTAGC
ATATGGGTCGGTGAGGTGGTGGAAAATATTTACCCCAGATCAATGTTCAGGCATCAACCGTTTTGTTGCA
GTGTTTGCAGTTCCATTACTCTCCTTCCATTTCATCTCCTCAAATGATCCATATGCTATGAACTACCACTTC
ATAGCTGCCGATTCTCTCCAGAAGGTAGTCATCCTCGTTGCCCTTTTCCTATGGCAAGCCTTCACCAAAC
ATGGCAATCTGGAGTGGATGATTACTCTCTTCTCTCTATCCACTCTCCCCAACACTCTTGTCATGGGGATC
CCCCTTTTAAAGGCTATGTACGGGGATTTCTCCGGCAGCCTTATGGTTCAAATTGTAGTTTTGCAGAGTGT
GATTTGGTACACTCTCATGCTTTTCATGTTTGAATATAGAGGAGCAAAGCTTCTGATCAACGACCAGTTCC
CGGAGACTGCTGGATCTATTACCTCTTTCCGTGTAGAGTCGGATGTTGTTTCACTTAATGGCCGCGAGCC
TCTACAAGCTGACGCTGAGATTGGTGATGACGGAAAACTCCACGTTGTGGTGAGAAGATCAAGTGCGTC
ATCAATGGTATCGTCCTTTAACAAATCACATGGACTGAATTCATTAACGTCCATGACACCCAGAGCCTCAA
ACCTTACCGGGGTAGAAATATACTCTGTCCAGTCATCCAGAGAGCCCACTCCCAGGGCTTCAAGCTTTAA
TCAAAACGATTTCTATGCCATGTTTGCAAGCAAGGCTCCAAGTCCCAACCATGGCTACAACAACAGCTTC
CAGGGGGCAGTTGGTGATGTTTTTTCGCTGCAATCTTCCAAAGGAGCAACACCAAGAACTTCAAACTTCG
ATGAAGAGATGCTCAAGGTTGCCAAGAGAAGGGGGGGAAGGAGCATGAGCGGCGAGTTGTATAACGGT
GGTGGTGGTGGTGGTGTTCCTTCTTATCCACCGCCCAATCCAATTTTTGCGGGGTCAACTAGCGGTGGC
TCTAAGAAGAAGGAAAGTGGCTCAATGCCTAACAAAGAGCTTCACATGTTTGTTTGGAGCTCTAGTGCTT
CTCCTGTGTCGGAAGGGAATCTAAGGCATGCAGTTAATAGAGCTGCCTCTACTGACTTTGACTCTTCTAA
ACCTACCTATCAACAAGAAAACGCTGCATCAAAAGCAATGCATGAGCTAATTGAAAATATGGGTAAAAGCA
GTGGGGGGGACAAGGATTTGGAGATATTAGAGGAGGGATCCAAGTTTCCAACAGGTGGATCTCCCTTGA
GTTGTCAGAAAAAGTTGAACATGGAAGAAGGCGAAGTGGCAAAGAAACAGCAGATGCCTCCTGCAAGTG
TGATGACAAGGCTTATTCTTATCATGGTTTGGAGGAAGCTCATTAGGAACCCTAACACATATTCCAGTCTT
TTCGGCTTGACTTGGTCCCTCATATCTTTCAGGTGGAACATTCAGATGCCAACAATTGTAAGTGGGTCGA

TAGCAATATTATCCGATGCTGGTCTGGGAATGGCAATGTTTAGTCTAGGCTTATTCATGGCATTACAACCC
AAGATCATAGCTTGTGGAAAATCTGTAGCAACATTTTCCATGGCTGTCAGGTTTTTGACTGGTCCCGCTGT
CATTGCTGCAACCTCCATAGCCATTGGTCTTCGTGGGGTTCTTTTGCATGTCGCTATCGTTCAGGCTGCC
CTTCCACAAGGAATAGTTCCATTTGTGTTTGCCAAGGAGTATAACGTTCATCCCGACATACTTAGCACTGC
GGTTATTTTCGGAATGTTGATTGCTCTGCCCATTACAATACTCTACTATGTGCTTCTTGGACTGTAA

>GhPIN8-2-A
ATGTCCTTCACTTTAACCCACAGACTCTGCCTTTCACCACCACAACGAACCCATCTCAGATTCACCTTCAA
ACCCCACACATCAAAACCCATAGTTTCTTCACCATCAAAACTCAGTTACCTTCCCATAGTCAGTTCTCTCA
GTCATAATTCTGAACACTTATCGGTCACTCAAGCAAAACCCAGATGGGAGAACATGTTATCGACTGCTGC
TAGCTTTTACCCTTTGTATGTGACTGTTGGTGGCATTGTGGCTTGTTTTAAGCCGTCTGCTTTTGCTTGGT
TCGTGGAGAGAGGACCTTCTTCATATAGCTTCTCGCTTGGGTTGATTATGTTGGCTATGGGTCTCACTTTA
GAACTCAAGGACTTGCTCAATTTGTTCACCCAAAGACCTCTTTCTATTCTATTTGGATGTGTTGCTCAGTA
CACAATTATGCCAACTTTTGCTATGGTTATTAGCAAAACTTTGGGGCTTTCACCATCTCTTTCTGTTGGTTT
GATTTTGCTAGGTTGTTGTCCTGGTGGTGCTGCATCCACTGTGGTGACCTTTATTGCACGAGGAGACGTT
TCGTTATCGACGGTAATGACGGTTTGCACTACTCTTGGTGCAGTGATCCTCACACCTCTTTTGACTGTGAT
TCTAGCAGGAACATATGTTCCTGTTGATGCTATTGGACTTTCCATCAGCACCCTGCAGGTGGTTGTGGCT
CCGGTTTTGCTCGGTTCTTACCTACAAAGCACATTTCCTTTGGTGGTGAAAATGATTACACCATTTGCTCC
TTTATTTGCCGTTTTACTTTCATCGCTACTTGCATGCAGCATTTTCTCGGGAAATGTCGTCCGTTTTAAGTC
CTCAATAGTTAATGCATCATTGGCTTCCGATGCCTCTTTAGTCTCACGACTCCAATCATTATTATCCGGAG
ACCTCGGTGCCGTAATACTTTCCGTGATGTTGCTACATTTTATCGGTTTCTTTGTAGGGTACATATCAGCA
GCCATTTGTAGATTCCGAGAAGCAGAGCGGCGAGCAATATCAATCGAGGTGCACATCTCCGAGTTTTATA
CCAAATCCCTTATGAACTCTTCATATATTGCATTACATGTGTTTGATGAAATCTCCCACTCAGGTTGGCAT
GCAAAATTCTTCATTAGGAGTGGTATTGGCAACAACTCATTTCACTTCTCCGGTGGTAGCGTTACCGGGC
GCCATGTCGGCAGTGATTATGAATATAATGGGTAG

>GhPIN8-1-D
ATGATTTCCCTGGCAGATGTTTATCATGTTATAGCAGCCACCGTGCCTTTATACTTTGCCATGATTTTAGC
CTACGTATCGGTGAAATGGTGGAAGCTGTTCACACCGGAACAATGCGCAGGCATCAACAAGTTCGTGGC
TAAATTCTCAATCCCACTCTTGTCTTTCCAAGTCATCTCCGAAAACAACCCTTACAAGATGAACCTCAAACT
CATACTTGCCGATTTCTTGCAAAAACTATTGGCTTTCCTTGTCTTGTTTGCCCTCACTAAGCTCAGTTCCC
GGGGTGGTTTGTCGTCCATCATAACCGGTCTCTCTTTATCCACCATGCCTAACACCTTAATCCTCGGAAT
CCCACTGTTGAGGGCTATGTATGGGGATAAATCAGCCACTCTTTTGGCTCAGATTGTCGTTCTACAAAGC
TTAATTTGGTATAATTTACTCTTGTTTTTGTTTGAGCTAAACGCTACCAAAGCTGCCTCTGAGATCACTGTT
GCAGTTTCACAAGCCTCAGGGGACGAAGAGGCCCCCGAAGAAGCACAAGGGAANGAGACACAAACGAG
AGCAAGGAAATCCAAGACAATGCTGATTTTCTTGACAGTGGGGAAGAAGCTAGTGGCGAACCCCAATAC
CCATGCTACCTTACTTGGTCTTATTTGGGCAAGCATTCAATTCAGGCTGTGGAACATTAAATTCCCCGCCA
TTGTTGAAAAATCAATAGCAATATTATCCAGTGGAGGACTTGGTATGGCAATGTTCAGCTTAGGTCTATTC
ATGGCATCACGGCCAAGCATAATAGCTTGTGGTATACGAATGGCGACAGTAGCCATGATCATGAAATTCA
TTGCTGGTCCAGCTCTAATGGCGGCCGCCTCCACCACTCTCGGACTTAAAGGCAAATTATTGAGAGTAGC
AATTGTACAGGCTGCTCTCCCTCAAGGAATCGTTCCATTTGTTTTCGCCAAAGAGTACAATGTTCATCCTG
ATATATTAAGCACAGGGGTAATCTTTGGTATGCTTATTGCCTTACCTGTAGCATTGGTCTACTATCTTCTTT
TAGCATTGTGA

>GhPIN9-A
ATGATTGGCATCAAGGATCTGTACAGTGTTCTGACTGCAGTTGTTCCTCTTTATGTCACCATGTTCTTGGC
TTATGGTTCAGTAAAATGGTGGAAAGTATTCACCCCCGAACAATGTGCCGGCATCAACAGATTCGTCGCC
ATCTTCGCCGTTCCGCTCCTCTCCTTCGAGTTCGTTTCGAGGATAAATCCTTACAAAATGGACCTCCTTTT
CCTTGCCGCTGATGGAGTCTCAAAAGTCCTGATATTGTTTGCTTTGCTTTGTTGGGCCAATTTCACAAAGA
GAGGCGGCCTGGACTGGTCTATCACAGTTTTCTCACTTTCCACACTCCCAAACACTCTTGTCATGGGGAT
TCCCCTACTGAAATCCATGTATGGGGATGACAAGGAATACCTAATGATTCAAGTTGTGGTGCTGCAATGC
ATAATCTGGTATACCCTATTACTGTTTTTGTTTGAATATAGAGAAACAAGAACTGAAGTCTTGAGCAAGTTC
AAGGAAAGTAGTGTTAGTTCTAAGAACTGTTGCAGTGAAGATGAAGTCATTAATGTTATTGCCACAACATC
ATCTAACCAGCAGCAGCAGCAGCAGCAGACGGCTCAAAATGCAAACAAGATTGCACCAGATCAATCTCA
GCGATTCAGGCCTATGGTAGCAGCATCGATGGAGGGTGAGGATGGGACAAGTACTACCTGTGAGAAGCA
GTCAGTGCAAGCTGGTCAAAGAGAAGAAAGCAGTAGTACTGCAAAGGGGGTTGAGAGTGTGAAGCAAGA
GAATGTCGAAAATGCTAGCATTCCCTCTTCTTTTTCTCCATCAATGCTCCTCAAAATATTGAGAAAAGTCTG
GCTCAAGCTTGTGAGGAACCCTAACTCTTACTCAAGTTTGCTGGGTCTTAGCTGGGCTTTAGTCTCTTGC
AGATGGGACATAAAGAAGCCCCAAATAATGGAAAATTCAGTCACGATATTGTCAAGTGCAGGTCTTGGGA
TGGCAATGTTTAGCCTTGGATTATTCATGGCTTTGCAACCAAGGATCATTGCCTGTGGAAAGAAGCTGGC

TTTATATGGGATGGTAGCTAGGTTTATAGCAGGACCGGCAGTAATGGCGATAGCATCCATCGCAGTTGGC
CTCAAAGGCACTACCTTAAAGTTGTCTATTGTACAAGCAGCATTGCCTCAAGGAATTGTCCCTTTCGTTTT
TTCTAGGGAGTATAATCTGCATCCTGATGGTGATATTTGGAATGATAGTTTCTCTGCCCATAACAATAGTA
TATTACATTGTGTTGGGTATTTGAGGAGTCATATTTGGAAGGATGAAAACATAAACATGAGGAACTCAGCA
GCATTGTTCAGAGAGATCCAGGGTTATGGTCAAAACCAGCAGCAGCAATATTCATATGTTTTTGTGCTGA
GTTGA

>GhPIN1-4-A
ATGATTAGTTGGAACGATCTTTACACCGTTTTAACGGCGGTGATCCCACTGTACGTTGCTATGATCTTGGC
TTACGGCTCCGTCCGTTGGTGGAAAATATTCACCCCCGACCAGTGCTCGGGTATCAACCGCTTTGTCGC
CATATTTGCCGTTCCTCTCTTGTCTTTCCACTTCATTTCCACCAATGACCCTTATGCCATGAACTTCAGGTT
CGTAGCAGCCGACACCCTTCAAAAGCTCATCATGCTCTTCGTTCTTGGATTGTGGACTAATTTAACAAGG
AACGGAAGCCTAGAATGGATGATCACCATTTTCTCTTTATCTACTCTCCCCAACACTCTAGTCATGGGCAT
TCCTCTTTTAATCGCTATGTACGGTCCCTACTCCGGGATGCTCATGGTCCAAGTCGTGGTTCTCCAGTGT
ATCATTTGGTACACCCTTCTTCTTTTCCTTTTTGAGTACCGCGGTGCCAAAATCCTCATCATGGAGCAGTT
TCCCGAAACTGCAGCTTCCATTGTTTCTTTCAAAGTTGATTCCGATGTGGTTTCACTTGACGGCCGCGACT
TCCTCGAAACCGACGCTGAAATCGGGGAAGACGGCAAGCTACACGTTAAGGTCAGGAAATCTAATGCTT
CCAGGAGGTCTTTGGGGCCTTGTTCCCTTCCTGCATTGACTCCCAGGCCTTCCAACCTCACCGGAGCTG
AAATCTACAGTTTGAGCTCTTCAAGGAACCCCACGCCAAGAGGTTCCAATTTCAACAACTCCGACTTTTAC
TCCATGATGGGTGTTCAAGGATTTCCTGCAAGACACTCCAATTTCGGTCCAGCTGATTTATACTCTGTTCA
ATCCTCTAGAGGACCTACTCCAAGGCCATCCAATTTTGAAGAAAACAATACAGTAATGTCTCCACGATTTG
GATTTTATCCAGCACAGACTGTTCCTTCATCCTACCCTGCTCCAAACCCTGAATTCTCATCCGTCACGAAG
AACGCTAAAGCTACCCAACAACAACAGCAGCAGCCAGTTCAACCACAGCAGCAGCCTAAGGAGAAGGAG
AATAATAAAGAGAATCATGATGCCAAGGAATTGCACATGTTTGTGTGGAGTTCAAGTGCCTCGCCGGTTT
CAGAAGGCGGAGGTCTCCATGTCTTTGGTGGTACAGATTTCGGAGCGTCCGAGCAATCTGGACGGTCTG
AGCAGGGTGCTAAAGAGATAAGGATGTTGGTCGCAGATCACCCTCAAAACGGGGAAAACAAAGGCATGA
CAGGCAGTGGTGACGTTAATGGAGAGGACTTTAGCTTTGCTGGAAGAGATGGGGAAGAGGAGAGAGAAA
AGGAAGGACCCAATGGTCTCAATAAGTTGGGGTCTAGTTCAACGGCCGAGTTGCACCCTAAAGCCGCCG
GAGGGCCTGAGTCTGGCGTAGGCAAACAAATGCCGCCGGCGAGTGTAATGACCCGCTTAATCTTGATCA
TGGTCTGGCGCAAGCTTATACGAAACCCCAACACATATTCCAGTCTCATCGGGCTGGTTTGGTCCCTAAT
TGCTTTCAGGTGGCATGTGAGTATGCCGAAAATAATAGAGAAGTCTATCTCCATCCTGTCAGATGCTGGA
CTAGGAATGGCCATGTTTAGCTTAGTAAGCGGTCCAGGGTCGGTGGGTATTGGTTGTGATAGACGCCAA
TTAGGCCACCAGTTAGTGAAGGTAGGTCTGTTTATGGCACTGCAACCCAAGATCATCGCTTGTGGGAACT
CTGTAGCTACATTTGCCATGGCCGTTAGGTTCTTAACCGGTCCGGCTGTCATGGCTGCCGCTTCGATTGC
AGTAGGGTTGCGCGGCACACTCCTCCGTGTCGCCATTGTTCAGGCGGCTCTGCCACAAGGAATTGTACC
ATTTGTGTTCGCCAAGGAATACAATGTCCACCCTGCAATTCTTAGCACTGCGGTTATCTTTGGGATGTTGA
TAGCATTACCAATAACGCTGGTGTACTACATTCTTCTAGGATTGTGA

>GhPIN6-D
ATGATAACAGGGGGGGATTTTTACAAGGTGATGTGTGCGATGGTTCCACTGTATTTCGCAATGATAGTAG
CGTACGGATCGGTGAAATGGTGGCGAATATTCAGCCCGGAGCAGTGTTCGGGGATCAACCGGTTCGTG
GCGGTTTTCGCGGTGCCGGTGTTGTCATTCCATTTCATAGCTCAAAACAACCCGTACCAGATGGACACCA
AGTTCATCATAGCGGACACGGTGTCCAAGGTGTTGGTCCTTGCTTTGCTTTCGGTTTGGGCTATCTTCTT
CCCTGGTGGCTCACTCGATTGGCTCATCACCCTTTTCTCCCTCGCCACTTTGCCCAACACTCTCGTCATG
GGCATCCCTTTGCTTAATGCTATGTATGGAGATTTCACTCAAAGTCTCATGGTTCAACTCGTTGTTCTTCA
ATGCATCATCTGGTACACACTATTGCTGTTCCTTTTCGAATACAGGGCGGCAACGCTCTTAATCAAAACCC
AGTTCCCGGGCCCCACAGCTGCCACCATTTCCAAATTTGAGCTCGACAACGACGTTATTTCATTGGACGG
TCGTGATCCTCTCCGTACAGAATCCGAAACCGACATCAACGGCCGTATTCGAGTCCGAATCAGGCGGTC
CACGTCATCGGCTCCGGAATCGGCTCTATCATCCTCCATTTGCCTCACCCCCAGAGCATCCAACCTCTCT
AACGCCGAAATATTTTCCGTTAACACCCCCGGCGGACCCAACAATAACGAGATTGTATTCTGTAACGGCG
ACTTGGGGTTCGGTTACCGTGCCGTTAGCCCCCGTTTGTCCGGGTATGCTTCGTCGGATGCTTACTCTTT
GCAGCCGACCCCAAGGGCTTCTAATTTTAATGAAATGGATGTGATTACTACGGCAGCCGGGAATACACCA
ATTTGGATGAGGTCTCCCGTGGCTGGTGGGAAAGTTTTCCGGCAGCCGTCTCCGGTGGTTCCTCCTACA
AAAATGGTTTGGGACTGTCAAGATGGTGGTGGGGATGATAATAGGCAGGGCTTCAAAGACCTTGGTGAG
AAGGAAATTAGCTTTAGAGACAACACGAAAATAACAGTGGCGGAGATGAATGGAGATGGGAAAGAAGGT
GAAGTGGGGAGCAAACAAGAAATGCCAAAGGCCATAGTGATGGTGAGACTCATTTTAATTGTTGTTGGAA
GGAAGCTTTCTCGAAACCCTAATACTTACTCAAGCATCTTAGGGCTTCTTTGGTCTCTCATCTCTTTCAAG
TGGAATGTAGGGATGCCGAGTTTGGTCAAGTATTCAATAAAAATAATCTCCGATGCTGGCCTTGGGATGG
CAATGTTCAGTTTAGGGTTATTTATGGCACTTCAGCCAAGGATTATTGCATGTGGAACAAAAAGAGCAACA
ATGGGGATGGTGATTCGTTTCCTGTGTGGCCCTGTAATAATGTCCACTGCATCGATAGCTCTGGGATTAA

GAGGGGCAAAACTACATGCAGCCATCGTACAGGCAGCCCTTCCCCAAGGTATCGTACCATTCGTCTTTG
CAAGAGAATACGGTTTACATCCCGACATATTAAGTACTGGGGTTATTTTTGGCATGTTGGTGTCATTGCCT
GTGACACTTCTGTATTACATACTTTTAGGCATATGA

>GhPIN1-3-D
ATGATCACTTTAACTGACTTCTACCATGTAATGACTGCAATGGTGCCACTTTACGTGGCCATGATTTTAGC
TTACGGCTCAGTGAAATGGTGGAAGATCTTCTCTCCTGGTCAGTGTTCGGGGATTAATCGCTTTGTTGCT
CTCTTTGCCGTCCCTCTCCTCTCTTTCCACTTCATCGCCTCTAACGACCCTTACGCTATGAACTTTCGTTT
CATAGCAGCTGATACCCTCCAAAAAGTTATGGTTCTTGGAATACTGGCAGTTTGGTCCAAGGTGAGCAAA
AGGGGTTGCTTGGAATGGACCATCACCCTATTTTCACTCTCTACTCTTCCCAACACTTTGGTGATGGGCA
TCCCTTTGCTGAAAGGAATGTACGGGGAGTTTTCAGGAAGCTTAATGGTGCAGATAGTTGTCCTTCAATG
CATTATTTGGTACACTTTGATGCTTTTCATGTTTGAATATAGAGGTGCCAAAATGCTGATATCTGAGCAATT
CCCCGACACTGCTGGCTCTATTGTTTCAATCCATGTAGACTCAGATATCATGTCACTCGATGGTCGGCAA
CCTTTAGAAACTGAAGCTGAGATCAAAGAAGATGGTAAGCTCCATGTTACTGTCAGGAAATCCAATGCCT
CAAGATCAGATATTTTCTCAAGAAGGTCCCAGGGTTTATCTTCAACAACTCCACGCCCTTCCAATCTGACC
AATGCTGAGATCTACTCGTTGCAATCATCAAGAAACCCAACACCAAGAGGCTCAAGCTTTAATCACACTG
ATTTCTACTCTATGATGGCTGGGGGACGCAACTCCAATTTCGGTGCTGCAGATGTTTACGGTTTGTCTGC
CTCTCGAGGACCAACTCCCAGACCATCCAATTATGAGGACGATGGTACAGGCATGGGTAAACCAAGGTT
CCATTACCACGCACAAGGTGGCGCAGGTGCAGCCCATTATCCGGCTCCTAATCCGGGTATGTTCTCTCC
CAATGGGTCTAAAGCTAATACAAAGAAGCCTAACGATCAGGCTCCGCAAAAGGCTGAAGATGGTGGCAG
GGATCTTCATATGTTTGTTTGGAGTTCAAGTGCTTCTCCTGTATCCGACGTCTTTGGTGGTGGTGGTGGC
CATGAATATGGAGCAAACGAGCAGAAAGAGGTTAGAGTGGCTGTCTCCCCAGGGAAAGCGGAAGGACAT
AGAAGAAATAACGAGGAGTACATGGAGAGAGAGGATTTCAGCTTTGGGAACCGAGGATTGGAACGAGAA
ATGACCAACAACCACGAAGGTGACAAAGTGGGGGATGGCAAGCCCAAAACTATGCCTCCTGCAAGTGTC
ATCACAAGGCTGATACTGATCATGGTTTGGAGAAAGCTTATCAGAAATCCCAACACTTATTCAAGCTTAAT
AGGACTCACTTGGTCTCTAATCTCATTCAGGTGGAATGTACAAATGCCTGCCATAATAGCCAAGTCCATTT
CCATACTGTCAGATGCAGGGCTTGGCATGGCCATGTTCAGTCTTGGTCTGTTCATGGCATTGCAACCGAG
GATCATAGCATGTGGAAATTCCGTTGCAGCTTTTGCCATGGGTGTGAGATTCCTTGCAGGTCCAGCTGTG
ATGGCAGCCGCTTCCATAGCTGTCGGACTCCGTGGCGTTCTCTTACACGTTGCCATTGTGCAGGCAGCT
CTCCCACAAGGCATTGTCCCCTTTGTCTTTGCCAAGGAATACAACCTACACCCTGATATTCTCAGCACTG
CTGTTATTTTTGGAATGCTAATAGCCTTGCCCATAACGCTTGTCTACTACATAGTATTGGGATTATGA

>GhPIN3-D
ATGATTAGTTGGAACGATCTTTACACCGTTTTAACGGCGGTGATCCCACTGTACGTTGCTATGATCTTGGC
TTACGGCTCCGTCCGTTGGTGGAAAATATTCACCCCCGACCAGTGCTCGGGTATCAACCGCTTTGTCGC
CATATTTGCCGTTCCTCTCTTGTCTTTCCACTTCATTTCCACCAATGACCCTTACGCCATGAACTTCAGGTT
CATAGCAGCCGACACCCTTCAAAAGCTCATCATGCTCTTCGTTCTTGGATTGTGGACTAATTTAACAAGGA
ACGGAAGCCTGGAATGGATGATCACCATTTTCTCTTTATCTACTCTCCCTAACACTCTAGTCATGGGTATT
CCTCTTTTAATCGCTATGTACGGTCCCTACTCCGGGATGCTCATGGTCCAAGTCGTGGTTCTCCAGTGTA
TCATTTGGTACACCCTTCTTCTTTTCCTTTTTGAGTACCGCGGTGCCAAAATCCTCATCATGGAGCAGTTT
CCCGAAACTGCAGCTTCCATTGTTTCTTTCAAAGTTGATTCCGATGTGGTTTCACTTGACGGCCGCGATTT
CCTCGAAACCGACGCTGAAATCGGGGAAGACGGCAAGCTACACGTTAAGGTCAGGAAATCTAATGCTTC
CAGGAGGTCTTTGGGGCCGTGTTCCCTTCCTGCATTGACTCCCAGGCCTTCCAACCTCACCGGTGCTGA
AATCTACAGTTTGAGCTCTTCAAGGAACCCCACGCCAAGAGGTTCCAATTTCAACAACTCCGATTTTTACT
CCATGATGGGTGTTCAAGGATTTCCTGCAAGACACTCCAATTTCGGTCCAGCTGATTTATACTCTGTTCAA
TCCTCTAGAGGACCTACTCCAAGGCCATCCAATTTTGAAGAAAACAATACAGTAATGTCTCCACGATTCG
GATTTTATCCAGCACAGACTGTTCCTTCATCCTACCCTGCTCCAAACCCTGAATTCTCATCCGTCACGAAG
AACGCTAAAGCTACCCAACAACAACAGCAGCAGCCTAGGGAGAAGGAGAATAATAAAGAGAATCATGAT
GCCAAGGAATTGCACATGTTTGTGTGGAGTTCAAGTGCTTCGCCGGTTTCAGAAGGCGGAGGTCTCCAT
GTCTTTGGTGGTACAGATTTCGGAGCGTCCGAACAATCTGGACGGTCTGAGCAGGGTGCTAAAGAGATA
AGGATGTTGGTCGCAGATCACCCTCAAAACGGGGAAAACAAAGGCATGGCAGGCAGTGGTGACGTTCAT
GGAGAGGACTTCAGCTTTGCTGGAAGAGATGGGGAAGAAGAGAGAGAAAAGGAAGGACCCAATGGTCT
CAATAAGTTGGGGTCTAGTTCAACGGCCGAGTTGCACCCTAAAGCCGCCGGAGGGCCGGAGTCTGGCG
TAGGCAAACAAATGCCGCCGGCAAGTGTAATGACGCGCTTAATCTTGATCATGGTCTGGCGCAAGCTTAT
CCGAAACCCCAATACATATTCCAGTCTCATCGGGCTGGTTTGGTCCCTAATTGCTTTCAGGTGGCATGTG
AGTATGCCGAAAATAATAGAGAAGTCTATCTCCATCCTGTCAGATGCTGGACTAGGAATGGCCATGTTTA
GCTTAGGTCTGTTTATGGCACTGCAACCCAAGATCATCGCTTGTGGGAACTCTGTAGCTACATTTGCCAT
GGCCGTTAGGTTCTTAACTGGTCCGGCTGTCATGGCTGCCGCTTCGATTGCAGTAGGGTTGCGCGGCAC
ACTCCTCCGTGTCGCCATCGTTCAGGCGGCTCTGCCACAAGGAATTGTACCATTTGTGTTCGCCAAGGAA

TACAATGTCCACCCTGCGATTCTTAGCACTGCGGTTATCTTTGGGATGTTGATAGCATTACCAATAACGCT
GGTGTACTATATTCTTCTAGGATTGTGA
