# Supplementary material for: The PIN gene family in cotton (Gossypium hirsutum): genome-wide identification and gene expression analyses during root development and abiotic stress responses
Source: BMC Genomics. 2017 Jul 3;18:507. doi: 10.1186/s12864-017-3901-5 (PMC5496148; doi:10.1186/s12864-017-3901-5)
Supplement: Supplementary file 4 — Multiple sequence alignment of the deduced amino acid sequences of predicted PINs from G. hirsutum. (PDF 2431 kb) [file 12864_2017_3901_MOESM4_ESM.pdf]

```
                           auxin efflux carrier (AEC) domain
GhPIN6-A    MITGGDFYKVMCAMVPLYFAMILAYGSVKWWRIFSPEQCSGINRFVAVFAVPVLSFHFIA   60
GhPIN6-D    MITGGDFYKVMCAMVPLYFAMIVAYGSVKWWRIFSPEQCSGINRFVAVFAVPVLSFHFIA   60
GhPIN1-3-A  MITLIDFYHVMTAMVPLYVAMILAYGSVKWWKIFSPGQCSGINRFVALFAVPLLSFHFIA   60
GhPIN1-3-D  MITLTDFYHVMTAMVPLYVAMILAYGSVKWWKIFSPGQCSGINRFVALFAVPLLSFHFIA   60
GhPIN1-1-D  MITLTDFYHVMTAMVPLYVAMILAYGSVKWWKIFTPDQCSGINRFVALFAVPLLSFHFIA   60
GhPIN1-2-D  MISITDFYHVMTAMVPLYVAMVLAYGSVKWWKIFTPDQCSGINRFVALFAVPLLSFHFIA   60
GhPIN1-4-D  MISATDLYHVLTAVVPLYVAMILAYGSVKWWKIFTPDQCSGINRFVALFAVPLLSFHFIS   60
GhPIN1-4-A  MISWNDLYTVLTAVIPLYVAMILAYGSVRWWKIFTPDQCSGINRFVAIFAVPLLSFHFIS   60
GhPIN3-D    MISWNDLYTVLTAVIPLYVAMILAYGSVRWWKIFTPDQCSGINRFVAIFAVPLLSFHFIS   60
GhPIN2-A    MITGKDIYDVLAAIVPLYIAMMLAYGSVRWWKIFTPDQCSGINRFVAVFAVPLLSFHFIS   60
GhPIN2-D    MITGKDIYDVLAAIVPLYIAMMLAYGSVRWWKIFTPDQCSGINRFVAVFAVPLLSFHFIS   60
GhPIN8-1-A  MISLADGYHVIAATVPLYFAMILAYVSVKWWKLFTPEQCAGINKFVAKFSIPLLSFQVIS   60
GhPIN8-1-D  MISLADVYHVIAATVPLYFAMILAYVSVKWWKLFTPEQCAGINKFVAKFSIPLLSFQVIS   60
GhPIN8-2-D  ------------------------------------------------------------    0
GhPIN9-A    MIGIKDLYSVLTAVVPLYVTMFLAYGSVKWWKVFTPEQCAGINRFVAIFAVPLLSFEFVS   60
GhPIN3-A    ------------------------------------------------------------    0
GhPIN8-2-A  --MSFTLTHRLCLSPPQRTHLRFTFKPHTSKPIVSSPSKLSYLPIVSSLSHNSEHLSVTQ   58

GhPIN6-A    SNNPYQMDTKFIIADTVSKVLVLALLSVWAIFFPGGSLDWLITLFSLATLPNTLVMGIPL  120
GhPIN6-D    SNNPYQMDTKFIIADTVSKVLVLALLSVWAIFFPGGSLDWLITLFSLATLPNTLVMGIPL  120
GhPIN1-3-A  SNDPYAMNFRFIAADTLQKVMVLGILAVWSKVSKRGCLEWTITLFSLSTLPNTLVMGIPL  120
GhPIN1-3-D  SNDPYAMNFRFIAADTLQKVMVLGILAVWSKVSKRGCLEWTITLFSLSTLPNTLVMGIPL  120
GhPIN1-1-D  SNNPYTMNFRFIAADTLQKIIVLVVLAIWSKVSKRGCLEWTITLFSLSTLPNTLVMGIPL  120
GhPIN1-2-D  SNDPYSMNFRFIAADTLQKLIVLAVLAVWTKVSKRGCLEWTITLFSLSTLPNTLVMGIPL  120
GhPIN1-4-D  TNNPYTMNLKFIAADTLQKLIVLVLLAIWSRTSSRGSLEWSITLFSLSTLPNTLVMGIPL  120
GhPIN1-4-A  TNDPYAMNFRFVAADTLQKLIMLFVLGLWTNLTRNGSLEWMITIFSLSTLPNTLVMGIPL  120
GhPIN3-D    TNDPYAMNFRFIAADTLQKLIMLFVLGLWTNLTRNGSLEWMITIFSLSTLPNTLVMGIPL  120
GhPIN2-A    SNDPYAMNYHFIAADSLQKVVILVALFLWQAFTKHGNLEWMITLFSLSTLPNTLVMGIPL  120
GhPIN2-D    SNDPYAMNYHFIAADSLQKVVILVALFLWQAFTKHGNLEWMITLFSLSTLPNTLVMGIPL  120
GhPIN8-1-A  ENNPYKMNLKLILADFLQKLLAFLVLFALTKLSSRGGLSSIITGLSLSTMPNTLILGIPL  120
GhPIN8-1-D  ENNPYKMNLKLILADFLQKLLAFLVLFALTKLSSRGGLSSIITGLSLSTMPNTLILGIPL  120
GhPIN8-2-D  ------MNIKLVLADFLQKVFAFVVLIAVGQLRSRGGEPSIITGFSLSTLPNTLILGIPL   54
GhPIN9-A    RINPYKMDLLFLAADGVSKVLILFALLCWANFTKRGGLDWSITVFSLSTLPNTLVMGIPL  120
GhPIN3-A    -------MLSTAASLYPLYVTVGGIVACFKPSAFAWFVERGPSSYSFSLGLIMLAMGITL   53
GhPIN8-2-A  AKPRWENMLSTAASFYPLYVTVGGIVACFKPSAFAWFVERGPSSYSFSLGLIMLAMGITL  118

GhPIN6-A    LNAMYGDFTQSLMVQLVVLQCIIWYTLLLFLFEYRAATLLIKTQFPGPTAATISKFELDN  180
GhPIN6-D    LNAMYGDFTQSLMVQLVVLQCIIWYTLLLFLFEYRAATLLIKTQFPGPTAATISKFELDN  180
GhPIN1-3-A  LKGMYGEFSGSLMVQIVVLQCIIWYTLMLFMFEYRGAKMLISEQFP-DTAGSIVSIHVDS  179
GhPIN1-3-D  LKGMYGEFSGSLMVQIVVLQCIIWYTLMLFMFEYRGAKMLISEQFP-DTAGSIVSIHVDS  179
GhPIN1-1-D  LKGMYGEFSGSLMVQIVVLQCIIWYTLMLFMFEYRGAKMLISEQFP-DTAGSIVSIHVDS  179
GhPIN1-2-D  LKGMYGDFSGSLMVQIVVLQCIIWYTLMLFMFEYRAAKMLISEQFP-GTAGSIVSIHVDS  179
GhPIN1-4-D  LKGMYGDYSGSLMVQIVVLQCIIWYTLMLFLFEYRGAKLLIAEQFP-DTAGSIISFKVDS  179
GhPIN1-4-A  LIAMYGPYSGMLMVQVVVLQCIIWYTLLLFLFEYRGAKILIMEQFP-ETAASIVSFKVDS  179
GhPIN3-D    LIAMYGPYSGMLMVQVVVLQCIIWYTLLLFLFEYRGAKILIMEQFP-ETAASIVSFKVDS  179
GhPIN2-A    LKAMYGDFSGSLMVQIVVLQSVIWYTLMLFMFEYRGAKLLINDQFP-ETAGSITSFRVES  179
GhPIN2-D    LKAMYGDFSGSLMVQIVVLQSVIWYTLMLFMFEYRGAKLLINDQFP-ETAGSITSFRVES  179
GhPIN8-1-A  LRAMYGDKSATLLAQIVVLQSLIWYNLLLFLFELN-------------------ATKAAS  161
GhPIN8-1-D  LRAMYGDKSATLLAQIVVLQSLIWYNLLLFLFELN-------------------ATKAAS  161
GhPIN8-2-D  LKAMYGDEPAGLLAQIVVLQSLIWYNLLLLMFEFN-------------------AAKPAS   95
GhPIN9-A    LKSMYGDDKEYLMIQVVVLQCIIWYTLLLFLFEYRETRTEVLSKFK-ESSVSSKNCCSED  179
GhPIN3-A    ELKDLLNLFTQRPLSILFGCVAQYTIMPTFAMIISKTLGLSPSLSVGLILLGCCPGGAAS  113
GhPIN8-2-A  ELKDLLNLFTQRPLSILFGCVAQYTIMPTFAMVISKTLGLSPSLSVGLILLGCCPGGAAS  178

GhPIN6-A    DVISLDGRDPLRTESETDINGRIRVRIRRSTSSAP------ESALSSSICLTPRASNLSN  234
GhPIN6-D    DVISLDGRDPLRTESETDINGRIRVRIRRSTSSAP------ESALSSSICLTPRASNLSN  234
GhPIN1-3-A  DIMSLDGRQPLETEAEIKEDGKLHVTVRKSNASRSDIFSRRSQGLSS---TTPRPSNLTN  236
GhPIN1-3-D  DIMSLDGRQPLETEAEIKEDGKLHVTVRKSNASRSDIFSRRSQGLSS---TTPRPSNLTN  236
GhPIN1-1-D  DIMSLDGRQPLETEAEIKDDGKLHVTVRKSNASRSDIFSRRSQGLSS---TTPRPSNLTN  236
GhPIN1-2-D  DVVSLDGRQPIETEAEIKEDGKLHITVRKSNASRSDIFSRRSHGFTS---TTPRPSNLTN  236
GhPIN1-4-D  DVLSLDGKEPLQTDAEVGDDGKLHVTVRKSTSSRSEIFSRRSHGLNSGMGLTPRPSNLTN  239
GhPIN1-4-A  DVVSLDGRDFLETDAEIGEDGKLHVKVRKSNASR------RSLGPCSLPALTPRPSNLTG  233
GhPIN3-D    DVVSLDGRDFLETDAEIGEDGKLHVKVRKSNASR------RSLGPCSLPALTPRPSNLTG  233
GhPIN2-A    DVVSLNGREPLQADAEIGDDGKLHVVVRRSSASSMVSSFSKSHGLNSLTSMTPRASNLTG  239
GhPIN2-D    DVVSLNGREPLQADAEIGDDGKLHVVVRRSSASSMVSSFNKSHGLNSLTSMTPRASNLTG  239
GhPIN8-1-A  ETTVAVSQASGGEEAPEEAQGKGGGEETQTRARKS-------------------------  198
GhPIN8-1-D  EITVAVSQASGDEEAPEEAQGX----ETQTRARKS-------------------------  192
GhPIN8-2-D  EITSTS-----EDIEDTEAQGKEREEEAETRASKF-------------------------  125
GhPIN9-A    EVINVIATTSSNQQQQQQTAQNANKIAPDQSQRFRPMVAASMEGEDGTSTTCEKQSVQA  239
GhPIN3-A    TVVTFIARGDVSLSTVMTVCTTLGAVILTPLLTMVLAG-------------TYVSIDAIG  160
GhPIN8-2-A  TVVTFIARGDVSLSTVMTVCTTLGAVILTPLLTVILAG-------------TYVPVDAIG  225
```

```
GhPIN6-A     ADIFSVNTPGGPNNNEIVFCNGDMGFGYRAVSPR-----------LSGYASSDAYSLQPT  283
GhPIN6-D     AEIFSVNTPGGPNNNEIVFCNGDLGFGYRAVSPR-----------LSGYASSDAYSLQPT  283
GhPIN1-3-A   AEIYSLQSSRNPTPRGSSFNHTDFYSMMAGG----RNSNF-----GAADVYGLSASRGPT  287
GhPIN1-3-D   AEIYSLQSSRNPTPRGSSFNHTDFYSMMAGG----RNSNF-----GAADVYGLSASRGPT  287
GhPIN1-1-D   AEIYSLQSSRNPTPRGSSFNHTDFYSMMAGG----RNSNF-----GSGDVYGMSASRGPT  287
GhPIN1-2-D   AEIYSLQSSRNPTPRGSSFNHTDFYSMMAGG----RNSNF-----GSADVYGLSASRGPT  287
GhPIN1-4-D   AEIYSLQSSRNPTPRGSSFNHTDFYSMVNGG----KNNVS-----PRQSNYGIGFDEENG  290
GhPIN1-4-A   AEIYSLSSSRNPTPRGSNFNNSDFYSMMGVQGFPARHSNF-----GPADLYSVQSSRGPT  288
GhPIN3-D     AEIYSLSSSRNPTPRGSNFNNSDFYSMMGVQGFPARHSNF-----GPADLYSVQSSRGPT  288
GhPIN2-A     VEIYSVQSSREPTPRASSFNQNDFYAMFASKAPSPKHGYTNSFQGAVGDVFSLQSSKGAT  299
GhPIN2-D     VEIYSVQSSREPTPRASSFNQNDFYAMFASKAPSPNHGYNNSFQGAVGDVFSLQSSKGAT  299
GhPIN8-1-A   ------------------------------------------------------------  196
GhPIN8-1-D   ------------------------------------------------------------  192
GhPIN8-2-D   ------------------------------------------------------------  125
GhPIN9-A     GQREESSSTAKGVESVKQENVENASIPSSFS-----------------------------  270
GhPIN3-A     LS ISTLQVVVAPVLIG-------------------------------------------  176
GhPIN8-2-A   LS ISTLQVVVAPVLLG-------------------------------------------  241

GhPIN6-A     PRASNFN-----------------------------------------------------  290
GhPIN6-D     PRASNFN-----------------------------------------------------  290
GhPIN1-3-A   PRPSNYEEDGTGMGKPRFHYHAQGG-------AGAAHYPAPNPGMFSPNGSK--------  332
GhPIN1-3-D   PRPSNYEDDGTGMGKPRFHYHAQGG-------AGAAHYPAPNPGMFSPNGSK--------  332
GhPIN1-1-D   PRPSNYEEDGS--GKPRFHYHGQS--------GVTGHYPAPNPGMFSPTGSK--------  329
GhPIN1-2-D   PRPSNYEEDGAAAGKPRFHYQAPGGGG---GGGGATHYPVPNPGMFSPTGSKPLGGGNA-  343
GhPIN1-4-D   VGMFGNNGIRS---------------------NGSSYPAPTSAGIFSPVTAP--------  321
GhPIN1-4-A   PRPSNFEENNTVMSPRFGFYPAQTVP---------SSYPAPNPEFSSVTKNAKATQQQQ-  338
GhPIN3-D     PRPSNFEENNTVMSPRFGFYPAQTVP---------SSYPAPNPEFSSVTKNAKATQQQ--  337
GhPIN2-A     PRTSNFDEEMLKVAKRRGGRSMSGELYNGGGGGGVPSYPPPNPIFAGSTSGGSKKKESGS  359
GhPIN2-D     PRTSNFDEEMLKVAKRRGGRSMSGELYNGGGGGGVPSYPPPNPIFAGSTSGGSKKKESGS  359
GhPIN8-1-A   ------------------------------------------------------------  196
GhPIN8-1-D   ------------------------------------------------------------  192
GhPIN8-2-D   ------------------------------------------------------------  125
GhPIN9-A     ------------------------------------------------------------  270
GhPIN3-A     ------------------------------------------------------------  176
GhPIN8-2-A   ------------------------------------------------------------  241

GhPIN6-A     --------------------------------------------EMDVITTAAGNTPIWM  306
GhPIN6-D     --------------------------------------------EMDVITTAAGNTPIWM  306
GhPIN1-3-A   ---------------------------------ANTKKPNDQAQQKAEDGGRDLHMFVWS  359
GhPIN1-3-D   ---------------------------------ANTKKPNDQAPQKAEDGGRDLHMFVWS  359
GhPIN1-1-D   ---------------------------------GVKKPNGQAHQKVEDGGGKDLHMFVWS  356
GhPIN1-2-D   -----------------------------N---ANAKRPNGHPQQKSEDGGRDLPVFVWS  371
GhPIN1-4-D   ----------------------------------------KKANGGGDGGGKDLHMFVWS  341
GhPIN1-4-A   -----------------------------QQPVQPQQQPKEKENNKENHDAKELHMFVWS  369
GhPIN3-D     -----------------------------------QQQPREKENNKENHDAKELHMFVWS  362
GhPIN2-A     MPNKELHMGGRSMSGELYNGGGVPSYPPPNPIFAGSTSGGSKKKEIGAMPNKELHMFVWS  419
GhPIN2-D     MPNK------------------------------------------------ELHMFVWS  371
GhPIN8-1-A   ------------------------------------------------------------  196
GhPIN8-1-D   ------------------------------------------------------------  192
GhPIN8-2-D   ------------------------------------------------------------  125
GhPIN9-A     ------------------------------------------------------------  270
GhPIN3-A     ------------------------------------------------------------  176
GhPIN8-2-A   ------------------------------------------------------------  241

GhPIN6-A     RSPVAGGKVFRQPSP-----------------VVPPTKMVWDCQDGGGDDNRQGFKDLGE  349
GhPIN6-D     RSPVAGGKVFRQPSP-----------------VVPPTKMVWDCQDGGGDDNRQGFKDLGE  349
GhPIN1-3-A   SSASPVSDVFGGGGG------------HEYGANEQKEVRVAVSPGKAEGHRENNEEYMER  407
GhPIN1-3-D   SSASPVSDVFGGGGG------------HEYGANEQKEVRVAVSPGKAEGHRRNNEEYMER  407
GhPIN1-1-D   SSASPVSDVFGGG--------------YEYGVTDQKEVRLAVSPGKVEGHRDNQEEFMER  402
GhPIN1-2-D   SSASPVSDVFGGTG-------------HDYGAADQKDVRLAVSPGKVEGHRENREEYMER  418
GhPIN1-4-D   SSASPVS---------------------------EGGIHVFKGGDYGNDHHHKDYDEYGR  374
GhPIN1-4-A   SSASPVSEGGGLHVFGGTDFGASEQSGRSEQGAKEIRMLVADHPQNGENKGMTGSGDVNG  429
GhPIN3-D     SSASPVSEGGGLHVFGGTDFGASEQSGRSEQGAKEIRMLVADHPQNGENKGMAGSGDVHG  422
GhPIN2-A     SSASPVSEGNLRHAVN-------------RAASTDFDSSKPTHQQENAASRAMHELIENM  466
GhPIN2-D     SSASPVSEGNLRHAVN-------------RAASTDFDSSKPTYQQENAASKAMHELIENM  418
GhPIN8-1-A   ------------------------------------------------------------  196
GhPIN8-1-D   ------------------------------------------------------------  192
GhPIN8-2-D   ------------------------------------------------------------  125
GhPIN9-A     ------------------------------------------------------------  270
GhPIN3-A     ------------------------------------------------------------  176
GhPIN8-2-A   ------------------------------------------------------------  241
```

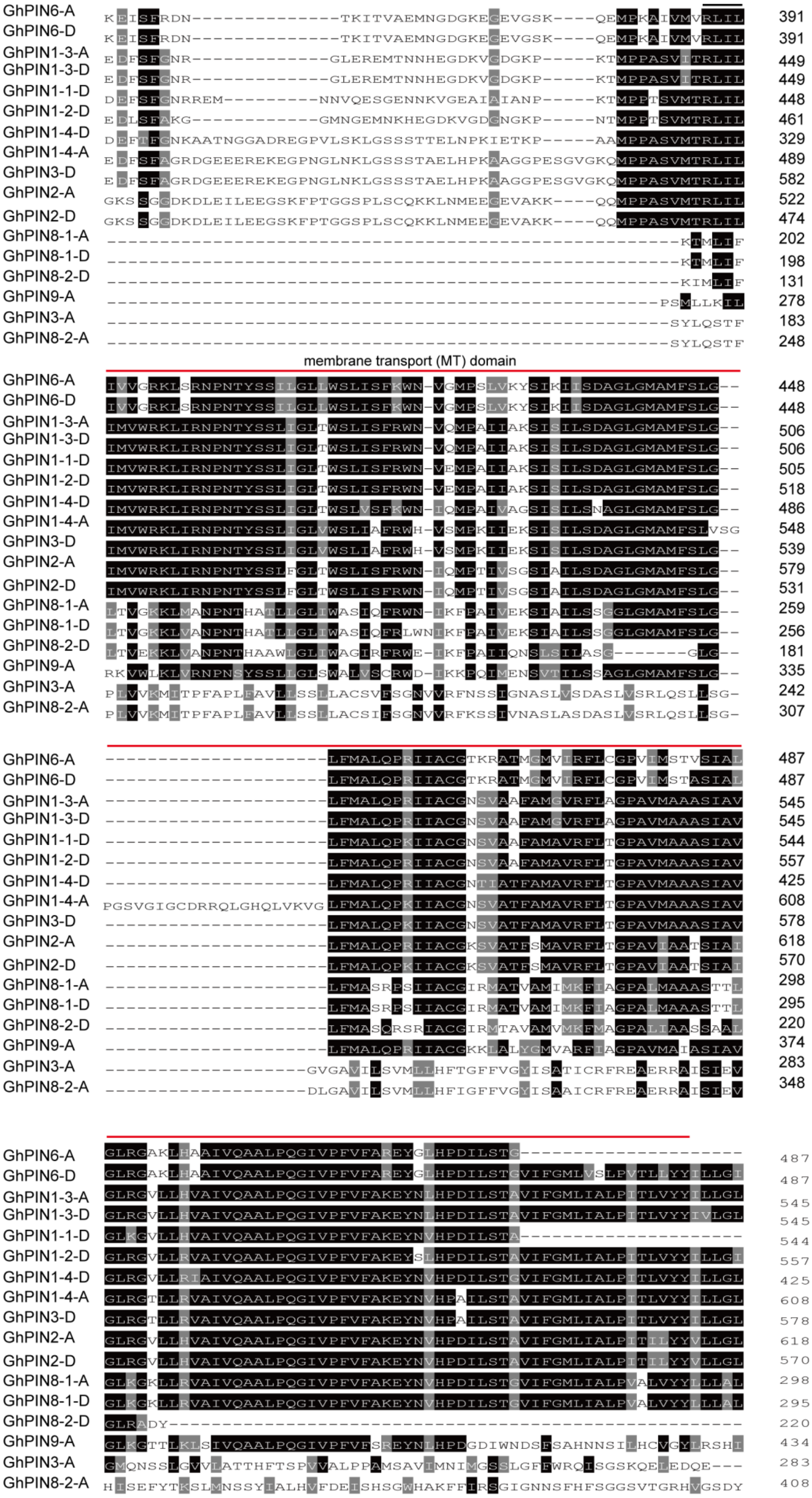
GhPIN6-A KEISFRDN--------------TKITVAEMNGDGKEGEVGSK----QEMPKAIVMVRLIL 391
GhPIN6-D KEISFRDN--------------TKITVAEMNGDGKEGEVGSK----QEMPKAIVMVRLIL 391
GhPIN1-3-A EDFSFGNR-------------GLEREMTNNHEGDKVGDGKP-----KTMPPASVITRLIL 449
GhPIN1-3-D EDFSFGNR-------------GLEREMTNNHEGDKVGDGKP-----KTMPPASVITRLIL 449
GhPIN1-1-D DEFSFGNRREM---------NNVQESGENNKVGEAIAIANP-----KTMPPTSVMTRLIL 448
GhPIN1-2-D EDLSFAKG------------GMNGEMNKHEGDKVGDGNGKP-----NTMPPTSVMTRLIL 461
GhPIN1-4-D DEFTFGNKAATNGGADREGPVLSKLGSSSTTELNPKIETKP-----AAMPPASVMTRLIL 329
GhPIN1-4-A EDFSFAGRDGEEEREKEGPNGLNKLGSSSTAELHPKAAGGPESGVGKQMPPASVMTRLIL 489
GhPIN3-D EDFSFAGRDGEEEREKEGPNGLNKLGSSSTAELHPKAAGGPESGVGKQMPPASVMTRLIL 582
GhPIN2-A GKSSGGDKDLEILEEGSKFPTGGSPLSCQKKLNMEEGEVAKK----QQMPPASVMTRLIL 522
GhPIN2-D GKSSGGDKDLEILEEGSKFPTGGSPLSCQKKLNMEEGEVAKK----QQMPPASVMTRLIL 474
GhPIN8-1-A --KTMLIF 202
GhPIN8-1-D --KTMLIF 198
GhPIN8-2-D --KIMLIF 131
GhPIN9-A --PSMLLKIL 278
GhPIN3-A ---SYLQSTF 183
GhPIN8-2-A ---SYLQSTF 248
membrane transport (MT) domain
GhPIN6-A IVVGRKLSRNPNTYSSILGLLWSLISFKWN-VGMPSLVKYSIKIISDAGLGMAMFSLG-- 448
GhPIN6-D IVVGRKLSRNPNTYSSILGLLWSLISFKWN-VGMPSLVKYSIKIISDAGLGMAMFSLG-- 448
GhPIN1-3-A IMVWRKLIRNPNTYSSLIGLTWSLISFRWN-VQMPAIIAKSISILSDAGLGMAMFSLG-- 506
GhPIN1-3-D IMVWRKLIRNPNTYSSLIGLTWSLISFRWN-VQMPAIIAKSISILSDAGLGMAMFSLG-- 506
GhPIN1-1-D IMVWRKLIRNPNTYSSLIGLTWSLISFRWN-VEMPAIIAKSISILSDAGLGMAMFSLG-- 505
GhPIN1-2-D IMVWRKLIRNPNTYSSLIGLTWSLISFRWN-VEMPAIIAKSISILSDAGLGMAMFSLG-- 518
GhPIN1-4-D IMVWRKLIRNPNTYSSLIGLTWSLVSFKWN-IQMPAIVAGSISILSNAGLGMAMFSLG-- 486
GhPIN1-4-A IMVWRKLIRNPNTYSSLIGLVWSLIAFRWH-VSMPKIIEKSISILSDAGLGMAMFSLVSG 548
GhPIN3-D IMVWRKLIRNPNTYSSLIGLVWSLIAFRWH-VSMPKIIEKSISILSDAGLGMAMFSLG-- 539
GhPIN2-A IMVWRKLIRNPNTYSSLFGLTWSLISFRWN-IQMPTIVSGSIAILSDAGLGMAMFSLG-- 579
GhPIN2-D IMVWRKLIRNPNTYSSLFGLTWSLISFRWN-IQMPTIVSGSIAILSDAGLGMAMFSLG-- 531
GhPIN8-1-A LTVGKKLMANPNTHATLLGLIWASIQFRWN-IKFPAIVEKSIAILSSGGLGMAMFSLG-- 259
GhPIN8-1-D LTVGKKLVANPNTHATLLGLIWASIQFRLWNIKFPAIVEKSIAILSSGGLGMAMFSLG-- 256
GhPIN8-2-D LTVEKKLVANPNTHAAWLGLIWAGIRFRWE-IKFPAIIQNSLSILASG-------GLG-- 181
GhPIN9-A RKVWLKLVRNPNSYSSLLGLSWALVSCRWD-IKKPQIMENSVTILSSAGLGMAMFSLG-- 335
GhPIN3-A PLVVKMITPFAPLFAVLLSSLLACSVFSGNVVRFNSSIGNASLVSDASLVSRLQSLLSG- 242
GhPIN8-2-A PLVVKMITPFAPLFAVLLSSLLACSIFSGNVVRFKSSIVNASLASDASLVSRLQSLLSG- 307
GhPIN6-A ---------------------LFMALQPRIIACGTKRATMGMVIRFLCGPVIMSTVSIAL 487
GhPIN6-D ---------------------LFMALQPRIIACGTKRATMGMVIRFLCGPVIMSTASIAL 487
GhPIN1-3-A ---------------------LFMALQPRIIACGNSVAAFAMGVRFLAGPAVMAAASIAV 545
GhPIN1-3-D ---------------------LFMALQPRIIACGNSVAAFAMGVRFLAGPAVMAAASIAV 545
GhPIN1-1-D ---------------------LFMALQPKIIACGNSVAAFAMAVRFLTGPAVMAAASIAV 544
GhPIN1-2-D ---------------------LFMALQPRIIACGNSVAAFAMAVRFLTGPAVMAAASIAV 557
GhPIN1-4-D ---------------------LFMALQPRIIACGNTIATFAMAVRFLTGPAVMAAASIAV 425
GhPIN1-4-A PGSVGIGCDRRQLGHQLVKVGLFMALQPKIIACGNSVATFAMAVRFLTGPAVMAAASIAV 608
GhPIN3-D ---------------------LFMALQPKIIACGNSVATFAMAVRFLTGPAVMAAASIAV 578
GhPIN2-A ---------------------LFMALQPRIIACGKSVATFSMAVRFLTGPAVIAATSIAI 618
GhPIN2-D ---------------------LFMALQPKIIACGKSVATFSMAVRFLTGPAVIAATSIAI 570
GhPIN8-1-A ---------------------LFMASRPSIIACGIRMATVAMIMKFIAGPALMAAASTTL 298
GhPIN8-1-D ---------------------LFMASRPSIIACGIRMATVAMIMKFIAGPALMAAASTTL 295
GhPIN8-2-D ---------------------LFMASQRSRIACGIRMTAVAMVMKFMAGPALIAASSAAL 220
GhPIN9-A ---------------------LFMALQPRIIACGKKLALYGMVARFIAGPAVMAIASIAV 374
GhPIN3-A -------------------GVGAVILSVMLLHFTGFFVGYISATICRFREAERRAISIEV 283
GhPIN8-2-A -------------------DLGAVILSVMLLHFIGFFVGYISAAICRFREAERRAISIEV 348
GhPIN6-A GLRGAKLHAAIVQAALPQGIVPFVFAREYGLHPDILSTG--------------------- 487
GhPIN6-D GLRGAKLHAAIVQAALPQGIVPFVFAREYGLHPDILSTGVIFGMLVSLPVTLLYYILLGI 487
GhPIN1-3-A GLRGVLLHVAIVQAALPQGIVPFVFAKEYNLHPDILSTAVIFGMLIALPITLVYYILLGL 545
GhPIN1-3-D GLRGVLLHVAIVQAALPQGIVPFVFAKEYNLHPDILSTAVIFGMLIALPITLVYYIVLGL 545
GhPIN1-1-D GLKGVLLHVAIVQAALPQGIVPFVFAKEYNVHPDILSTA--------------------- 544
GhPIN1-2-D GLRGVLLRVAIVQAALPQGIVPFVFAKEYSLHPDILSTAVIFGMLIALPITLVYYILLGI 557
GhPIN1-4-D GLRGVLLRIAIVQAALPQGIVPFVFAKEYNVHPDILSTGVIFGMLIALPITLVYYILLGL 425
GhPIN1-4-A GLRGTLLRVAIVQAALPQGIVPFVFAKEYNVHPAILSTAVIFGMLIALPITLVYYILLGL 608
GhPIN3-D GLRGTLLRVAIVQAALPQGIVPFVFAKEYNVHPAILSTAVIFGMLIALPITLVYYILLGL 578
GhPIN2-A GLRGVLLHVAIVQAALPQGIVPFVFAKEYNVHPDILSTAVIFGMLIALPITILYYVLLGL 618
GhPIN2-D GLRGVLLHVAIVQAALPQGIVPFVFAKEYNVHPDILSTAVIFGMLIALPITILYYVLLGL 570
GhPIN8-1-A GLKGKLLRVAIVQAALPQGIVPFVFAKEYNVHPDILSTGVIFGMLIALPVALVYYLLLAL 298
GhPIN8-1-D GLKGKLLRVAIVQAALPQGIVPFVFAKEYNVHPDILSTGVIFGMLIALPVALVYYLLLAL 295
GhPIN8-2-D GLRADY-- 220
GhPIN9-A GLKGTTLKLSIVQAALPQGIVPFVFSREYNLHPDGDIWNDSFSAHNNSILHCVGYLRSHI 434
GhPIN3-A GMQNSSLGVVLATTHFTSPVVALPPAMSAVIMNIMGSSLGFFWRQISGSKQELEDQE--- 283
GhPIN8-2-A HISEFYTKSLMNSSYIALHVFDEISHSGWHAKFFIRSGIGNNSFHFSGGSVTGRHVGSDY 408

```
GhPIN6-A     ------------------------------------  487
GhPIN6-D     ------------------------------------  487
GhPIN1-3-A   ------------------------------------  545
GhPIN1-3-D   ------------------------------------  545
GhPIN1-1-D   ------------------------------------  544
GhPIN1-2-D   ------------------------------------  557
GhPIN1-4-D   ------------------------------------  425
GhPIN1-4-A   ------------------------------------  608
GhPIN3-D     ------------------------------------  578
GhPIN2-A     ------------------------------------  618
GhPIN2-D     ------------------------------------  570
GhPIN8-1-A   ------------------------------------  298
GhPIN8-1-D   ------------------------------------  295
GhPIN8-2-D   ------------------------------------  220
GhPIN9-A     WKDENINMRNSAALFREIQGYGQNQQQQYSYVFVLS  470
GhPIN3-A     ------------------------------------  283
GhPIN8-2-A   EYNG--------------------------------  412
```

Figure S1. Multiple sequence alignment of the deduced amino acid sequences of predicted PINs from *G. hirsutum*.

The graphical view of the alignment was generated by BioEdit using ClustalW. Black under lines indicates the auxin efflux carrier (ACE) domain and red under lines highlight the membrane transport (MT) domain.
