# Supplementary material for: The PIN gene family in cotton (Gossypium hirsutum): genome-wide identification and gene expression analyses during root development and abiotic stress responses
Source: BMC Genomics. 2017 Jul 3;18:507. doi: 10.1186/s12864-017-3901-5 (PMC5496148; doi:10.1186/s12864-017-3901-5)
Supplement: Supplementary file 5 — The number of PIN genes identified across 7 plant species. (PDF 89 kb) [file 12864_2017_3901_MOESM5_ESM.pdf]

Table S3. The number of PIN genes identified across 7 plant species.

Species	Long PIN			PIN9	Short PIN			Total
	PIN1	PIN2	PIN3/4/7		PIN6	PIN5	PIN8	
G. hirsutum-BGI	6	2	2	1	2	0	4	17
G. hirsutum-NBI	7	2	2	1	2	0	2	16
G. barbadense-NBI	6	2	8	1	3	0	2	22
G. barbadense-Esquel	6	1	4	1	2	0	1	15
A. thaliana	1	1	3	0	1	1	1	8
G. arboreum	4	1	2	1	1	2	1	12
G. raimondii	4	1	1	1	1	1	1	10
