# Supplementary material for: The PIN gene family in cotton (Gossypium hirsutum): genome-wide identification and gene expression analyses during root development and abiotic stress responses
Source: BMC Genomics. 2017 Jul 3;18:507. doi: 10.1186/s12864-017-3901-5 (PMC5496148; doi:10.1186/s12864-017-3901-5)
Supplement: Supplementary file 6 — Measurements of lateral root number of three-week-old G. hirsutum and G. arboreum seedlings. (PDF 100 kb) [file 12864_2017_3901_MOESM6_ESM.pdf]

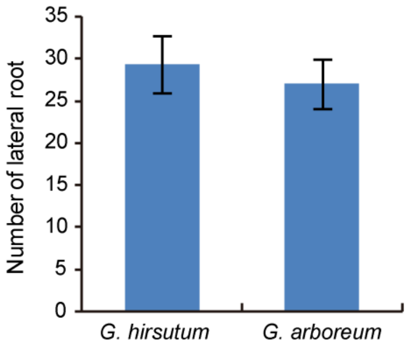

Figure S2. Measurements of lateral root number of three-week-old *G. hirsutum* and *G. arboreum* seedlings.

Statistical analyses were obtained from three replicates with a total of 30 seedlings for each species. The lateral root number in this figure represents the average number of all investigated lateral roots. Data is represented as means ± SE.
